# Epimutations driven by RNAi or heterochromatin evoke antifungal drug resistance in human fungal pathogens

**DOI:** 10.1101/2025.06.17.660219

**Published:** 2025-06-30

**Authors:** Ye-Eun Son, Joseph Heitman

**Affiliations:** 1 Department of Molecular Genetics and Microbiology, Duke University Medical Center, Durham, NC 27710, USA

**Keywords:** *Mucor*, Epigenetic resistance, RNA silencing, Histone modification, *In vivo* stability

## Abstract

Antimicrobial resistance is a global health threat emerging through microbe adaptation, driven by genetic variation, genome plasticity or epigenetic regulation. This study investigates how the *Mucor circinelloides* species complex adapts to the antifungal natural product FK506. In *Mucor bainieri*, most resistant strains exhibit unstable phenotypes without genetic changes. Approximately ~50% of FK506-resistant isolates acquire resistance via RNAi-dependent epimutation, where small interfering RNAs (siRNAs) silence *fkbA* transcription. The remaining isolates undergo heterochromatin-mediated silencing via H3K9 methylation and siRNAs spreading, repressing *fkbA* and neighboring genes. One isolate retained only heterochromatin marks without detectable siRNAs. A similar mechanism operates in *Mucor atramentarius*, where FK506 resistance is mediated by ectopic heterochromatin associated with siRNA. Strikingly, heterochromatin-based epimutation inheritance remains stable following *in vivo* infection. These findings reveal that antifungal resistance can arise through distinct, heritable epigenetic pathways involving RNAi, heterochromatin, or both highlighting adaptive strategies employed by ubiquitous eukaryotic microbial pathogens infecting humans.

## Introduction

Fungi are ubiquitous eukaryotic organisms that occupy diverse ecological niches. While many fungi contribute to food production, pharmaceutical development, and biotechnology, others are pathogens capable of infecting a wide range of hosts including humans [1]. The global burden of fungal infections has risen dramatically, now accounting for an estimated two million deaths annually [2, 3]. Despite this growing threat, therapeutic options remain limited due to a restricted antifungal arsenal, inadequate diagnostics, and an alarming rise in antifungal resistance. The widespread use of antifungal agents in agriculture and healthcare, combined with environmental pressures such as climate change, has intensified selective pressure on fungal populations, driving the emergence of fungal antimicrobial resistance (fAMR) [4–7].

Fungi employ both genetic and epigenetic strategies to adapt to antifungal stress [8, 9]. Genetic changes include point mutations (missense/nonsense) and insertions and deletions (indels) in drug target genes (e.g., *erg11*, *cyp51*, *fks1*), as well as largescale chromosomal alterations such as copy number variation and aneuploidy. These changes confer resistance in several human fungal pathogens including *Candida albicans*, *Cryptococcus neoformans*, and *Aspergillus fumigatus* [10, 11]. In contrast, epigenetic modifications enable non-Mendelian inheritance of resistance without altering the underlying DNA sequence. These include processes such as DNA methylation, RNA interference (RNAi), and ectopic heterochromatin [12].

In *Mucor circinelloides*, RNAi-dependent silencing of *fkbA*—which encodes FKBP12, the cellular target of FK506 (tacrolimus)—confers transient FK506 resistance that reverts in the absence of drug pressure [13]. RNAi-mediated silencing of *pyrF* or *pyrG* prevents conversion of 5-fluoroorotic acid (5-FOA) into its toxic form, leading to transient 5-FOA resistance in *Mucor circinelloides* [14]. In addition, heterochromatin-driven gene silencing has been implicated in stress adaptation. In *Schizosaccharomyces pombe*, exposure to caffeine, an environmental stressor, induces the formation of histone 3 lysine 9 dimethylation (H3K9me2)-enriched heterochromatin islands that silence genes and promote survival without genetic mutation [15].

Mucormycosis is a life-threatening infection caused by Mucorales species including *Mucor* and *Rhizopus* [16]. Spores enter the host via inhalation, traumatic injury, or the rhino-orbital-cerebral route, often resulting in rapid dissemination, especially in immunocompromised individuals with conditions such as diabetes mellitus, malignancy, malnutrition, organ transplantation, or trauma [17, 18]. The mortality rate of mucormycosis ranges from 50 to 90%, and incidence surged globally during the COVID-19 pandemic, with over 3,000 deaths reported in India alone [19–21]. Recognizing the severity of mucormycosis, the World Health Organization (WHO) recently designated Mucorales as a high-priority fungal group in the Fungal Priority Pathogens List to drive research and therapeutic development [9, 22]. Nonetheless, both intrinsic and acquired antifungal resistance in *Mucor* spp. continue to undermine treatment efficacy, underscoring the urgent need to define resistance mechanisms.

The *Mucor circinelloides* complex (MCC) comprises at least 16 phylogenetic species (PS), most of which are predominantly isolated from clinical cases and primarily involved in infections within Mucorales [23]. Prior studies in *M. lusitanicus* and *M. circinelloides* have identified both Mendelian and epigenetic mechanisms of FK506 resistance, including RNAi-mediated silencing of *fkbA* in the absence of mutations [13, 24]. However, whether such epigenetic regulation is conserved across other MCC species—or whether alternative mechanisms exist—remains unclear. In this study, we demonstrate that antifungal FK506 resistance in MCC arises through diverse genetic and epigenetic mechanisms, including RNAi-mediated and heterochromatin-mediated silencing. Notably, RNAi and heterochromatin functionally interact to modulate gene expression and confer FK506 resistance in some epimutants. Additionally, we demonstrate that FK506 resistance mediated by H3K9me2 heterochromatin is stably inherited after *in vivo* infection, providing new insights into the durability and clinical relevance of epigenetic antifungal resistance.

## Results

### Screening of *Mucor circinelloides* species complex (MCC) reveals isolates resistant to FK506

Previous studies reported the phylogenetic classification of the MCC and divided the group into 16 phylogenetic species (PS) based on a polyphasic approach that included multi-locus sequence analyses, as well as morphological and physiological characterization [23]. RNAi-mediated epigenetic gene silencing has been shown to play a key role in FK506 resistance in *Mucor lusitanicus* (PS10) and *M. circinelloides* (PS14 and PS15) [13, 24]. In this study, we screened multiple species to isolate strains resistant to FK506, a calcineurin inhibitor known to affect the hyphae-to-yeast transition, a process that has been linked to virulence in *Mucor* [25], and rapamycin which inhibits TOR and blocks nutrient-stimulated growth [26]. Following the strategy outlined in Fig 1A, spores were collected and point-inoculated on YPD media with or without FK506, and the emergence of resistant colonies was monitored. Resistant colonies were observed in *Mucor janssenii* (PS1), *Mucor bainieri* (PS3), *Mucor atramentarius* (PS6), and *Mucor pseudocircinelloides* (PS13), as well as in the previously reported *M. lusitanicus* (PS10) and *M. circinelloides* (PS14 and PS15) (**S1 Fig**).

To investigate the underlying mechanisms of resistance, we selected FK506-resistant isolates from *M. janssenii, M. bainieri,* and *M. atramentarius* for further analysis (Fig 1B). The *fkbA* gene, three genes encoding calcineurin catalytic A (*cnaA*~*C*) subunits, and the calcineurin regulatory B subunit (*cnbR*) gene were amplified and analyzed by Sanger sequencing. As summarized (Table 1), four out of ten FK506-resistant isolates from *M. janssenii* exhibited large deletions spanning the *fkbA* locus. An additional five isolates carried large insertions (~4 to 5 kb) in the *fkbA* promoter, nonsense mutations within *fkbA* exons, or point mutations at splice sites. In one isolate, a point mutation in the calcineurin A subunit gene (*cnaA*) was associated with FK506 resistance but retained rapamycin sensitivity. Thus, in *M. janssenii*, all ten isolates exhibited Mendelian mutations. In contrast, analysis of ten FK506-resistant isolates from *M. bainieri* revealed only one isolate with a nonsense mutation in *fkbA*, while the remaining nine exhibited no detectable sequence changes in *fkbA*, or the genes encoding calcineurin A or B subunits, suggesting a possible epigenetic regulation (Table 1). Similarly, in *M. atramentarius*, among ten FK506-resistant isolates, three harbored nonsense mutations in *fkbA*, one contained a synonymous mutation, two had splice-site point mutations, one had a start-loss mutation, and one had a point mutation in calcineurin B subunit (*cnbR*) (Table 1). Notably, two isolates showed no genetic alterations in any of the sequenced genes.

These results indicate that all ten FK506-resistant isolates from *M. janssenii* are Mendelian mutants. In *M. bainieri*, one isolate exhibited a Mendelian mutation, while the remaining nine were identified as candidate epimutants. In *M. atramentarius*, eight isolates carried Mendelian mutations, and two were classified as candidate epimutants.

### FK506-resistant epimutants display reversible phenotypes characterized by silenced *fkbA* transcription and FKBP12 protein expression

**Fig 2.**
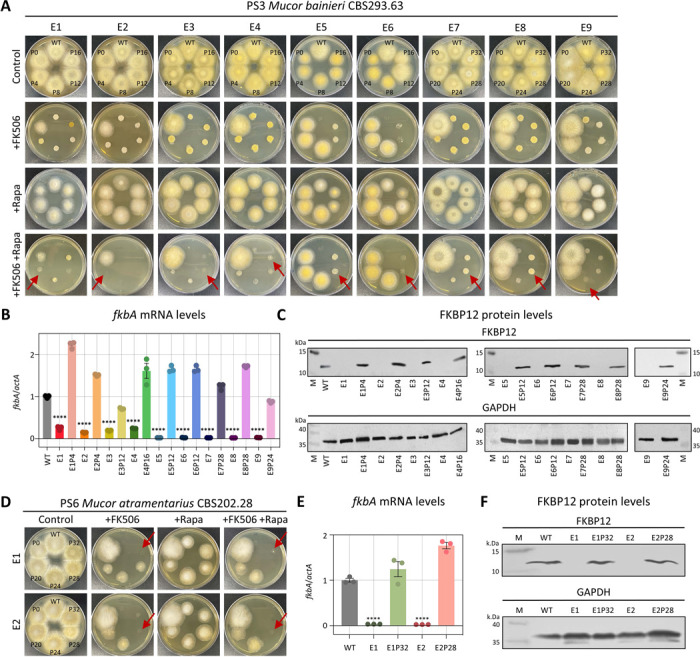
FK506-resistant epimutants exhibit silencing of *fkbA* mRNA and FKBP12 protein expression and are unstable. **(A, D)** Phenotypic reversion of FK506-resistant epimutant strains following serial passage without drug selection in *M. bainieri* (A) or *M. atramentarius* (D). Red arrows indicate the points at which epimutants reverted to the WT phenotype. FK506 was utilized at 1 μg/mL and rapamycin at 100 ng/mL. WT, wild-type; P, number of passages. **(B, E)** Quantification of the *fkbA* mRNA expression in *M. bainieri* (B) *or M. atramentarius* (E). Error bars represent mean ± SEM (n = 3). Statistical significance: ****p ≤ 0.0001. **(C, F)** Western blot analysis of FKBP12 protein expression in *M. bainieri* (C) or *M. atramentarius* (F). PS3: E1–E9, epimutants; E1P4–E9P24, revertants. PS6: E1 and E2, epimutants; E1P32 and E2P28, revertants.

For FK506-resistant isolates where no DNA sequence alterations were detected in the FK506 target gene *fkbA* or the calcineurin genes, we hypothesized that resistance might be regulated by epigenetic mechanisms. Because epimutations are typically associated with reversible phenotypic plasticity [14], we tested whether FK506 resistance would be lost after successive subculturing on drug-free medium. In fact, FK506 resistance was progressively lost following serial passages in the absence of selection pressure (Fig 2A, 2D and Table 2). Resistant strains exhibited a hyphal morphology and were capable of sporulation on FK506-containing media, whereas both wild-type (WT) and revertant strains displayed a yeast-like morphology when exposed to FK506 treatment (**S2A, S2D Fig**). Additionally, in *M. bainieri*, phenotypic reversion occurred after an average of 15.5 passages, whereas in *M. atramentarius*, reversion required approximately 30 passages (Fig 2A and 2D). Next, considering that epigenetic alterations inhibit target gene expression without changes in the underlying nucleotide sequence, we assayed *fkbA* transcript and FKBP12 protein levels. As shown in Fig 2B and 2E, the *fkbA* mRNA levels in all FK506-resistant strains were significantly reduced compared to WT and were restored in all reverted strains. Consistently, FKBP12 protein was undetectable in resistant strains and was restored in reverted epimutant isolates (Fig 2C and 2F). These results suggest that drug-resistant strains lacking genetic changes represent epimutants, and that resistance reversion varies both between and within species.

### Small RNAs confer FK506 resistance in *M. bainieri*

To determine which epigenetic mechanisms are involved in FK506 resistance in *M. bainieri*, we first evaluated the potential role of DNA methylation. Whole-genome sequencing data revealed the absence of canonical DNA 5-methylcytosine methyltransferases (DNMT1, DIM-2, RID-1, DNMT4, and DNMT5), consistent with a previous study [27], suggesting that DNA methylation is not involved in FK506 resistance.

**Fig 3.**
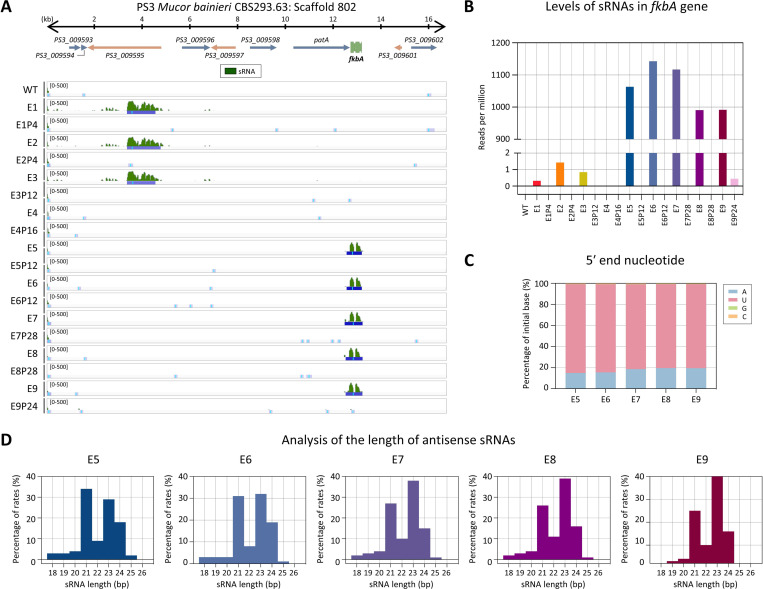
Small RNAs mediate FK506 resistance in *M. bainieri*. **(A)** Antisense small RNA coverage mapped across the *fkbA* gene and its neighboring loci. The peaks shown below represent significant enrichment peaks, validated by MACS3. **(B)** Small RNA abundance (RPM: reads per million) mapped to the *fkbA* gene. **(C)** 5′-end nucleotide preference of small RNAs associated with the *fkbA* gene. **(D)** Size distribution of small RNAs mapped to the *fkbA* gene. WT, wild-type; E1–E9, epimutants; E1P4–E9P24, revertants.

We next examined the involvement of RNAi by performing sRNA sequencing. As shown in Fig 3A, small antisense RNAs mapping to the *fkbA* locus were detected in most FK506-resistant epimutants but were absent in WT and revertant strains. The distribution pattern of sRNAs varied across epimutants: E5–E9 exhibited strong and specific accumulation of sRNAs at the *fkbA* locus. Additional analysis revealed that *fkbA*-targeting sRNAs in E5–E9 averaged approximately 1,000 reads per million (RPM) (Fig 3B). The 5′ nucleotide of these sRNAs was predominantly uracil (~82%; Fig 3C), and their length distribution peaked at 21–24 nucleotides (nt) (Fig 3D), consistent with the general features of Argonaute-loaded sRNAs [13, 28]. These results indicate that E5–E9 epimutants are RNAi-dependent epimutants, in which FK506 resistance is mediated by *fkbA*-targeting antisense sRNAs that silence *fkbA* gene expression.

In contrast to E5–E9, which exhibited abundant siRNAs mapping to *fkbA*, isolates E1–E4 lacked detectable *fkbA*-targeting siRNAs (Fig 3A and Fig 3B). However, E1–E3 displayed siRNAs mapping to neighboring genes (**S3 Fig**), with a strong 5′ uridine bias and predominant lengths of 21–24 nt. Strikingly, E4 lacked detectable siRNAs at both *fkbA* and its neighboring loci. To determine whether sRNAs spreading at *fkbA*-adjacent loci was associated with transcriptional repression, we performed reverse transcription quantitative PCR (RT-qPCR). As shown in **S3O Fig**, E1–E4 exhibited significant downregulation of genes flanking *fkbA*. However, no significant changes were observed in the expression of *fkbA*-neighboring genes in E7–E9. Meanwhile several genes (e.g., *PS3_009596*, *PS3_009598*) showed significantly altered expression in E5–E6, but these alterations were not associated with sRNA abundance.

### Heterochromatin marks, along with RNAi, contribute to epigenetic resistance in *M. bainieri*

**Fig 4.**
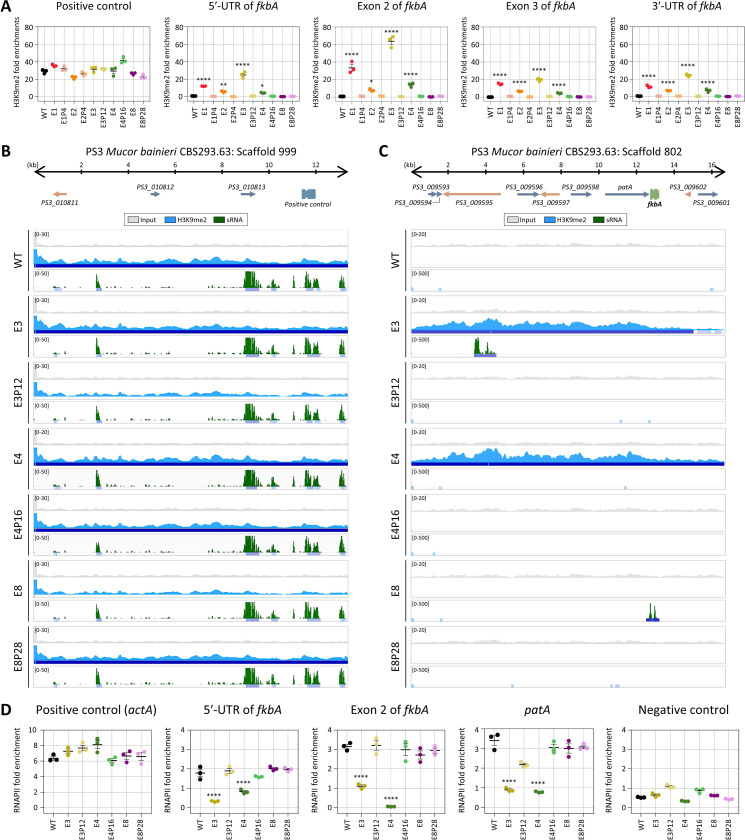
Heterochromatin marks underlie epigenetic FK506 resistance in *M. bainieri*. **(A)** ChIP-qPCR analysis of H3K9me2 enrichment at the positive control region (an siRNA-enriched locus) and *fkbA* locus (targeting the 5′-UTR, exonic regions, and 3′-UTR). Error bars represent mean ± SEM (n = 3). Statistical significance: *p ≤ 0.05, **p ≤ 0.01; ****p ≤ 0.0001. **(B, C)** ChIP-seq coverage mapped across the positive control loci (B) or *fkbA* and its neighboring loci (C). The peaks shown below represent significant enrichment peaks, validated by MACS3. **(D)** ChIP-qPCR analysis of RNA polymerase II enrichment at the positive control region (*actA*), *fkbA* locus (targeting the 5′-UTR and exonic region), a *fkbA*-neighboring gene, and negative control region (an siRNA-enriched locus marked by H3K9me2). Statistical significance: ****p ≤ 0.0001. WT, wild-type; E1–E8, epimutants; E1P4–E8P28, revertants

Small RNA spreading has been closely linked to heterochromatin formation and epigenetic gene silencing. Small RNAs targeting specific loci can initiate or reinforce histone modifications, particularly H3K9 methylation [15, 29]. To determine whether histone-mediated epigenetic regulation contributes to FK506 resistance in *M. bainieri*, we conducted chromatin immunoprecipitation (ChIP) to assess the abundance of histone H3 lysine 9 dimethylation (H3K9me2) in the following epimutants: E1–E3, which exhibit sRNA spreading; E4, which lacks detectable sRNAs but shows transcriptional repression of *fkbA* and neighboring genes; and E8, a canonical RNAi-dependent *fkbA* epimutant. ChIP-qPCR was conducted across the *fkbA* locus, targeting the 5′ UTR, two exonic regions, and the 3′ UTR. H3K9me2 enrichment at the positive control region, which was enriched with small RNAs, was comparable across all strains (Fig 4A). H3K9me2 enrichment at *fkbA* locus was negligible in WT, E8, and its revertant E8P28. In contrast, significant enrichment of H3K9me2 was observed in epimutants E1–E4 and was lost upon reversion, suggesting that H3K9me2 is associated with unstable antifungal drug resistance.

To further investigate heterochromatin-based epimutational gene silencing, we performed H3K9me2 ChIP-sequencing on the epimutant strains E3, E4, and E8. All strains exhibited similar patterns and levels of H3K9me2 and sRNA at positive control loci, supporting the specificity of the assay (Fig 4B). However, in E3 and E4, H3K9me2 signals extended beyond the *fkbA* locus into neighboring regions, suggesting heterochromatin spreading (Fig 4C). Notably, while E3 showed both H3K9me2 and sRNA spreading, E4 displayed H3K9me2 enrichment without detectable sRNAs. In contrast, the RNAi-dependent epimutant E8 exhibited strong sRNA accumulation specifically at the *fkbA* locus but lacked H3K9me2 enrichment. Taken together, these findings reveal three distinct epigenetic strategies underlying FK506 resistance in *M. bainieri* as summarized in Table 3: RNAi-associated heterochromatin-dependent mechanisms (Group 1), heterochromatin-dependent only (Group 2), and RNAi-dependent only (Group 3). Interestingly, Group 1 epimutants required fewer passages (average 6.67) for phenotypic reversion compared to epimutants in Group 2 (16.00) or Group 3 (18.80), suggesting that dual-layered epigenetic regulation may promote a more readily reversible resistance state.

Next, we sought to determine whether gene silencing in these epimutants is regulated at the pre- or post-transcriptional level. Generally, heterochromatin formation compacts chromatin structure, preventing RNA polymerase II (RNAPII) from accessing DNA and thereby blocking transcription initiation, which is classified as pre-transcriptional regulation [30]. In contrast, RNAi pathways typically involve sRNA that target and degrade transcripts after transcription, representing post-transcriptional regulation [31]. To evaluate the transcriptional status of the *fkbA* locus, we performed RNAPII ChIP-qPCR assays. As shown in Fig 4D, RNAPII occupancy at a positive control region, *actA*, was comparable across all strains, while occupancy at the negative control region, which was enriched for sRNAs and marked by H3K9me2, was negligible in all strains. In contrast, the 5′-UTR, exon, and adjacent regions of *fkbA,* where heterochromatin spreading was observed, was significantly reduced in E3 and E4 compared to WT, the RNAi-dependent epimutant E8, and revertant strains. These findings indicate that gene silencing in Group 1 (RNAi-associated heterochromatin-dependent mechanisms) and Group 2 (heterochromatin-dependent only) occurs at the pre-transcriptional level, whereas silencing in Group 3 (RNAi-dependent only) operates through post-transcriptional regulation.

### Heterochromatin mediates FK506 resistance in *M. atramentarius*

**Fig 5.**
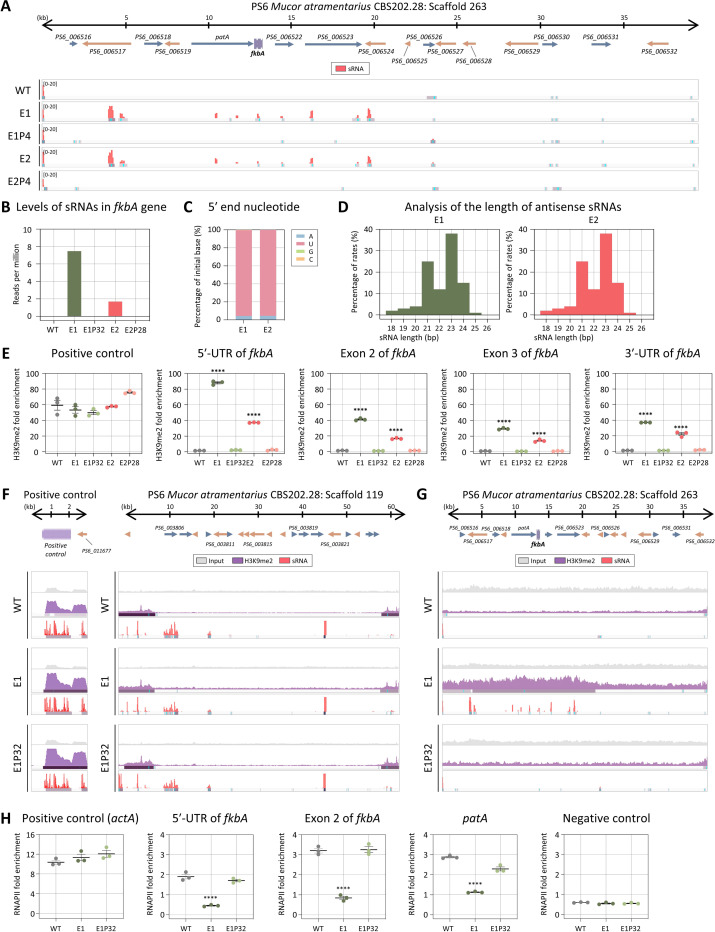
Heterochromatin-associated FK506 resistance is conserved in *M. atramentarius*. **(A)** Antisense small RNA coverage mapped across the *fkbA* gene and its neighboring loci. The peaks shown below represent significant enrichment peaks, validated by MACS3. **(B)** Small RNA abundance (RPM: reads per million) mapped to the *fkbA* gene. **(C)** 5′-end nucleotide preference of small RNAs associated with the *fkbA* gene. **(D)** Size distribution of small RNAs mapped to the *fkbA* gene. **(E)** ChIP-qPCR analysis of H3K9me2 enrichment at the positive control region (an siRNA-enriched locus) and *fkbA* locus (targeting the 5′-UTR, exonic regions, and 3′-UTR). Error bars represent mean ± SEM (n = 3). Statistical significance: ****p ≤ 0.0001. **(F, G)** ChIP-seq coverage mapped across the positive control loci (F) or *fkbA* and its neighboring loci (G). The peaks shown below represent significant enrichment peaks, validated by MACS3. **(H)** ChIP-qPCR analysis of RNA polymerase II enrichment at the positive control region (*actA*), *fkbA* locus (targeting the 5′-UTR and exonic region), a *fkbA*-neighboring gene, and negative control region (an siRNA-enriched locus marked by H3K9me2). Statistical significance: ****p ≤ 0.0001. WT, wild-type; E1 and E2, epimutants; E1P32 and E2P28, revertants.

We next assessed whether RNAi- and heterochromatin-based resistance mechanisms identified in *M. bainieri* also operate in *M. atramentarius*, another member of the *Mucor circinelloides* species complex. To evaluate the potential involvement of RNAi, we performed sRNA sequencing. As shown in Fig 5A, antisense sRNA spreading was observed across the *fkbA* locus and its flanking regions. Although sRNA abundance at *fkbA* itself was too low (Fig 5B), the sRNAs exhibited a strong 5′ uridine bias (Fig 5C) and a length distribution peaking at 21–24 nt (Fig 5D), consistent with canonical RNAi-derived sRNAs.

Given that the pattern of sRNA spreading resembled that observed in heterochromatin-associated epimutants in *M. bainieri* (**S4 Fig**), we next examined histone modification. ChIP-qPCR revealed significant H3K9me2 enrichment at the *fkbA* locus in the epimutants (Fig 5E). While H3K9me2 enrichment at the positive control locus, which is enriched with small RNAs, was comparable across all strains, expanded H3K9me2 signals at *fkbA* and its neighboring genes, accompanied by sRNA spreading, were strikingly observed exclusively in the epimutant (Fig 5F and 5G). Consistently, RT-qPCR revealed significantly reduced expression of *fkbA*-neighboring genes in the epimutants compared to WT and revertant strains (**S5 Fig**). Furthermore, consistent with observations in *M. bainieri*, we observed a significant decrease in RNAPII enrichment at the 5′-UTR, exon, and neighboring regions of *fkbA* in the *M. atramentarius* epimutant, indicating that heterochromatin-mediated gene silencing in *M. atramentarius* is associated with pre-transcriptional regulation (Fig 5H). Collectively, these results support the conservation of heterochromatin-mediated FK506 resistance in *M. atramentarius*, reinforcing its role as a general epigenetic mechanism across the *Mucor circinelloides* complex.

### Antifungal resistance mediated by heterochromatin is stably inherited following *in vivo* passage

**Fig 6.**
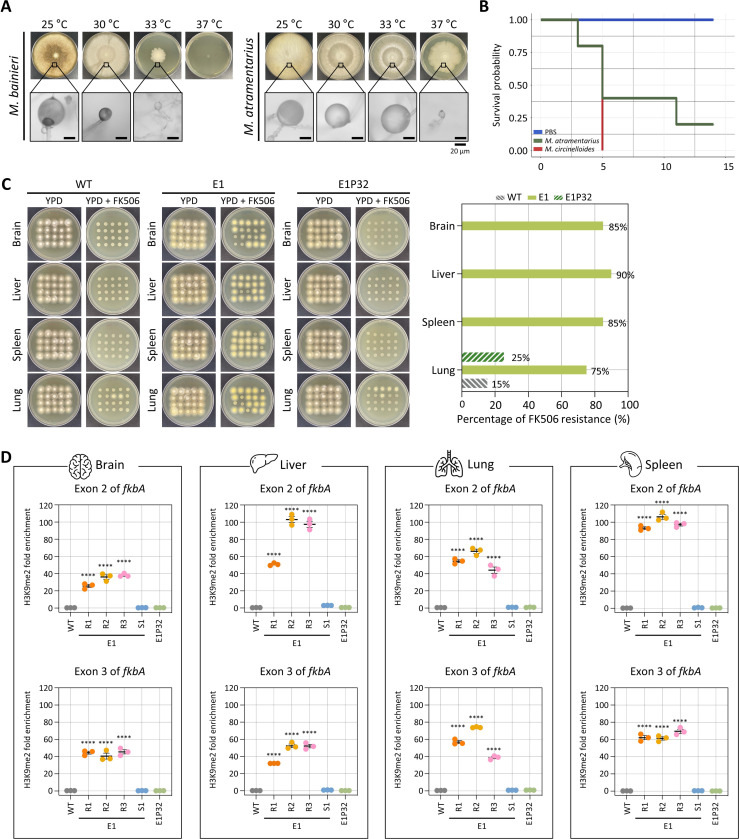
Inherited heterochromatin-mediated antifungal resistance persists after *in vivo* passage. **(A)** Fungal growth of *M. bainieri* and *M. atramentarius* under different temperature conditions. Microscopic images show enlarged sporangia (scale bar = 20 μm). **(B)** Survival rates of mice infected with *M. atramentarius*. *Mucor circinelloides* and PBS served as positive and negative controls, respectively. The log-rank test revealed a statistically significant difference in survival between groups (*M. atramentarius* vs PBS, *p* = 0.014). **(C)** Fungal colonies recovered from four different organs following *in vivo* infection with wild-type (WT), epimutant (E1), and revertant (E1P32) strains. The bar plot (right) shows the percentage of FK506-resistant colonies from each organ after passage. **(D)** ChIP-qPCR analysis of H3K9me2 enrichment at the *fkbA* locus following *in vivo* passage, targeting two exonic regions. Error bars represent mean ± SEM (n = 3). Statistical significance: ****p ≤ 0.0001. WT, wild-type; E1R1–E1R3, FK506-resistant epimutants; E1S1, FK506-sensitive epimutant; E1P32, revertant.

Previous studies reported that RNAi-mediated antifungal resistance can be stably transmitted following *in vivo* passage [32]. To investigate whether heterochromatin-mediated resistance also persists through cell division and stress during host infection, we first examined the maximum growth temperature of *M. bainieri* and *M. atramentarius*. *M. atramentarius* was able to grow at 37 °C, suggesting potential pathogenicity, whereas *M. bainieri* failed to grow under the same condition (Fig 6A). To further assess pathogenicity of *M. atramentarius*, we infected fungal spores (1 × 10^6^) via retro-orbital injection in a murine model. Mice infected with *M. circinelloides*, a known pathogen causing mucormycosis, exhibited a significant reduction in survival (Fig 6B). Notably, infection with *M. atramentarius* also led to a marked decrease in survival, demonstrating its pathogenic potential.

Based on these findings, we infected immunosuppressed mice with equal number of spores from WT, FK506-resistant epimutant (E1), and revertant (E1P32) strains of *M. atramentarius*. On day 4 post-infection, brain, liver, spleen, and lung tissues were harvested to assess fungal dissemination. Fungal colonization levels were comparable across strains, with no significant differences observed (**S6A Fig**). Subsequently, when fungal colonies recovered from each organ were tested on FK506-containing media, resistance was retained in colonies derived from epimutant-infected mice (over 80% across organs) in contrast to those derived from WT and revertant strains (Fig 6C). Interestingly, a few colonies recovered from the lungs of WT- and revertant-infected mice also exhibited FK506 resistance. Given that heterochromatin mediates FK506 resistance in epimutants, we next asked whether this epigenetic state persists following *in vivo* passage. To address this, we performed ChIP-qPCR on fungal colonies recovered from four organs. FK506-resistant epimutants exhibited robust H3K9me2 enrichment not only at exonic regions but also across the *fkbA* promoter and terminator (Fig 6D and **S6B**). By contrast, H3K9me2 levels were minimal or undetectable in WT and revertant strains. These findings indicate that heterochromatin-mediated antifungal resistance is largely stable during host infection, likely maintained through mitotic inheritance of H3K9 methylation marks.

## Discussion

Fungal antimicrobial resistance (fAMR) represents an urgent and growing global health threat, jeopardizing human and animal health, agriculture, and food security [7]. Although antifungal drugs are widely used to mitigate this burden, the remarkable adaptability of fungi to chemical stressors has rendered them increasingly difficult to control. Paradoxically, dual antifungal therapies intended to improve treatment outcomes may instead accelerate the evolution of multidrug-resistant strains [33–35]. A deeper understanding of the mechanisms driving fAMR is critical for developing effective interventions across clinical and environmental settings.

**Fig 7.**
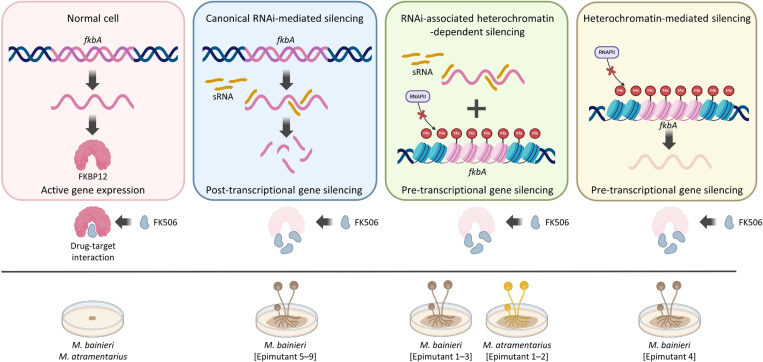
Schematic overview of epigenetic genetic silencing contributing to antifungal resistance in *Mucor* species. *fkbA* expression enables FKBP12 production and FK506 sensitivity (yeast-like morphology). Epigenetic silencing of *fkbA* via RNAi or heterochromatin leads to FK506 resistance and hyphal growth. Generated with BioRender.com.

FK506 targets FKBP12, a conserved protein that when bound to FK506 inhibits the calcineurin pathway—an essential regulator of fungal growth, morphogenesis, stress responses, and virulence [36]. In *M*. *circinelloides*, FK506 enforces a morphological shift from hyphae to yeast-like cells, thereby attenuating pathogenicity [25]. Here, we show that FK506 regulates morphology across the *Mucor circinelloides* complex (**S1 Fig**), and that resistance arises via both Mendelian and non-Mendelian mechanisms (Fig 1 and Table1). Our findings expand current models of antifungal resistance by revealing that RNAi- or heterochromatin-mediated epimutations represent alternative, and sometimes independent, pathways. These mechanisms may function separately or synergistically to silence *fkbA*, operating through either post-transcriptional or pre-transcriptional regulation to confer FK506 resistance, and resulting in resistance phenotypes with varying degrees of stability, specificity, and reversibility (Fig 7).

The interplay between RNAi and histone methylation raises key questions about the sequence and causality of these silencing events. Preexisting heterochromatin can initiate RNAi, as seen in centromeres and transposable elements in *S. pombe* and *Arabidopsis thaliana*, where transcripts from heterochromatic loci are processed into siRNAs that reinforce silencing [37]. In *S. pombe*, chaperone proteins like Hsp90 stabilize the RNA-induced transcriptional silencing (RITS) complex, which recruits Clr4 to nascent transcripts, facilitating heterochromatin formation [38]. Conversely, euchromatic loci may sporadically generate double-stranded RNA, initiating RNAi and leading to heterochromatin deposition via a positive feedback loop[39]. However, in *M. circinelloides*, RNAi and heterochromatin formation have been shown to function independently in repressing transposable elements, contributing to genome stability [27]. Interestingly, in our study, we identified FK506-resistant epimutants with varying combinations of siRNA accumulation and H3K9me2 enrichment at the *fkbA* locus (Fig 4). These findings support two non-exclusive models: (1) RNAi-driven initiation of heterochromatin, or (2) heterochromatin-facilitated RNAi. Given that the *fkbA* gene is located in a euchromatic region, the RNAi-first model appears more plausible, although chromatin-initiated RNAi cannot be excluded. Future studies analyzing pathway-specific mutants will be critical to determine the directionality and mechanistic coupling of these epigenetic processes.

Maintaining epigenetic silencing is energetically demanding. The RNAi pathway requires ATP for the cleavage of double-stranded RNA by Dicer and the unwinding of siRNA duplexes [40]. Histone methylation also relies on ATP-driven production of S-adenosylmethionine (SAM), a cofactor of histone methyltransferase, and ATP-dependent chromatin remodeling, which is essential for stable heterochromatin maintenance [41, 42]. These processes reflect a substantial metabolic cost to the cell. Notably, epimutants governed by both RNAi and heterochromatin-dependent silencing exhibited faster phenotypic reversion than those regulated by a single mechanism (Table 3). This reduced stability may stem from the cumulative energetic burden of maintaining multiple ATP-intensive pathways. The associated metabolic cost could impose selective pressure favoring reversion, thereby restoring cellular fitness. Alternatively, redundancy or incomplete reinforcement between the two silencing systems may inherently destabilize the dual-layered epimutation, rendering it more prone to reversion than resistance maintained by either pathway alone.

Torres-Garcia et al. demonstrated that external stressors such as caffeine induce phenotypic plasticity via Clr4/H3K9me “read-write” systems in *S. pombe*, underscoring the adaptive function of heterochromatin in non-pathogenic fungi [15]. Our findings extend this concept by providing direct evidence that H3K9 methylation contributes to FK506 resistance in dimorphic *Mucor* species. Notably, this mechanism operates in pathogenic potential *M. bainieri* and *M. atramentarius* species, suggesting that heterochromatin-mediated resistance is conserved across diverse fungal taxa, spanning both unicellular yeasts and multinuclear hyphal species. While RNAi-dependent antifungal resistance has been shown to be rapidly induced and reversed following *in vivo* passage in a murine model [32], the *in vivo* stability of heterochromatin-dependent epimutants has not been previously characterized. Here, we demonstrate that a heterochromatin-dependent epimutant remains stable *in vivo* (Fig 6). The persistence, even in the absence of antifungal pressure, raises the possibility that epigenetically resistant strains could silently disseminate in clinical settings, complicating diagnostics and treatment.

In nature, diverse microorganisms, including bacteria, viruses, and fungi, interact continuously, forming complex ecological networks [43–46]. Soil systems are nutrient-rich environments that support a vast diversity of microorganisms, which in turn contribute to essential ecological functions such as nutrient cycling and organic matter decomposition [47, 48]. *Streptomyces* is one of the most widely distributed and ecologically significant genera within the phylum Actinobacteria, renowned for its remarkable ability to produce a wide variety of bioactive secondary metabolites, including antifungal compounds[49]. Here, we used FK506, a natural antifungal compound that is produced by several *Streptomyces* species, such as *Streptomyces hygroscopicus* and *Streptomyces tsukubaensis* [50, 51]. Mucorales represent a major fungal order of soil-dwelling saprophytic fungi [52], and thus the ecological coexistence of *Streptomyces* and *Mucor* in soil raises the possibility that natural occurrence of the antifungal natural product FK506 may impose a selective pressure on fungal populations. This pressure may promote the emergence of epigenetic resistance, even in the absence of anthropogenic drug use, by enabling fungi to flexibly modulate gene expression in response to environmental antifungal stress, thereby enhancing their survival and competitive fitness in chemically dynamic ecosystems.

In summary, our study extends RNAi-dependent gene silencing to additional *Mucor* species and uncovers that heterochromatin-mediated silencing also drives antifungal resistance. These findings underscore the remarkable epigenetic plasticity of fungal epigenomes and highlight adaptive strategies that enable reversible, yet durable, resistance. Targeting RNAi and chromatin-modifying pathways may thus represent a promising therapeutic strategy against fAMR.

## Material and methods

### Fungal strains, media and growth conditions

*Mucor* strains studied here are described in **S1 Table**. *Mucor* strains were grown on liquid or solid yeast extract peptone dextrose (YPD; BD, Difco, Franklin Lakes, NJ, USA) at room temperature. To isolate FK506-/rapamycin-resistant strains, 10^5^ spores were inoculated onto solid YPD, supplemented with antifungal drug 1 μg/mL of FK506 (Astellas Pharma, Chuo, Tokyo, Japan) and 100 ng/ml of rapamycin (API Chem, Hangzhou, Zhejiang, Chian) and incubated for 3, 5, 7 days [13]. To test the stability of FK506-resistant strains, spores of the resistant strains were collected and inoculated in liquid YPD without any drugs overnight [14]. After several rounds of subculturing, the passaged cells were spotted onto YPD or YPD with FK506 to confirm phenotypic plasticity.

### Sanger sequencing analysis

*Mucor* strains were cultured on YPD medium for three days and gDNA was extracted from with the Tissue & Cell Lysis Buffer (Lucigen, Middleton, WI, USA) and MPC Protein Precipitation Reagent (Lucigen). The promoter, open reading frame, and terminator regions of the *fkbA* gene were amplified by polymerase chain reaction (PCR) using primers listed in **S2 Table**. The purified PCR products were sequenced and analyzed against the reference *fkbA* sequence.

### RNA extraction and quantitative PCR (qPCR) analysis

Total RNA was extracted from Mucor cells cultured on YPD, with or without FK506, at room temperature. After overnight lyophilization, each sample was homogenized in a FastPrep bead-beater (MP Biomedicals, Santa Ana, CA, USA) with 0.2 mL of zirconia-silica beads (BioSpec Products, Bartlesville, OK, USA). Total RNA was then purified with the miRNeasy Mini Kit (Qiagen, Hilden, Germany) [53]. The isolated RNA was treated with DNase and subsequently employed for cDNA synthesis with the Maxima H Minus cDNA Synthesis Master Mix (ThermoFisher, Waltham, MA, USA). Real-time PCR was performed with PowerUp SYBR Green Master Mix (ThermoFisher) on a QuantStudio 3 qPCR system (ThermoFisher) in triplicate. The primers for qPCR are listed in **S2 Table**. Gene expression was normalized to *actA*, relative fold expression was calculated using 2^−ΔΔCt^ method.

### Small RNA (sRNA) sequencing

sRNA libraries were constructed with the QIAseq miRNA Library Kit (Qiagen) and sequenced on an NovaSeq X platform (Illumina) by 75-bp single end. Raw reads were trimmed with adaptor sequence (5′-GTTCAGAGTTCTACAGTCCGACGATC-3′ and 5′-AACTGTAGGCACCATCAA-3′) utilizing BBDuk. Filtered reads were then mapped to the reference genomes (*M. bainieri* CBS293.63; PRJNA1277536 or *M. atramentarius* CBS202.28; PRJNA1277544) using BBMap [54]. The resulting SAM files were converted to BAM files, and antisense reads were sorted using SAMtools (v1.21) [55]. BAM files were converted to TDF files using IGV tools with a window size of 20 bp and the mean window function. The data was subsequently visualized in the Integrative Genome Viewer (IGV v2.16.0) [56]. The Y-axis reflects the average read coverage per 20 bp window. Peak calling was performed with MACS3 using a fold-enrichment range between 5 and 50. sRNA-seq experiments were performed with one biological sample per strain.

### Western blot

Each *Mucor* strain was cultivated in YPD, with or without FK506, at room temperature for three days. After overnight lyophilization, each sample was homogenized using a FastPrep bead-beater (MP Biomedicals) with 0.2 mL of zirconia-silica beads (BioSpec Products). Pierce RIPA buffer (ThermoFisher) containing a protease inhibitor tablet (Roche) was then added, and each sample was further disrupted with a FastPrep bead-beater. The samples were incubated on ice for at least 30 min. Cell extracts were centrifuged at 14,000 RPM, 4°C for 10 min, and the supernatants were quantified with the Pierce BCA Protein Assay Kit (ThermoFisher). Next, 20 μg of each sample was mixed with Novex Tris-Glycine SDS Sample Buffer (Invitrogen, Waltham, MA, USA) and boiled at 85°C for 5 min. After briefly cooling, the samples were separated by SDS-PAGE, transferred to a PVDF membrane, and incubated overnight at 4°C with an anti-FKBP12 antibody (Invitrogen) or an anti-GAPDH antibody (Proteintech, Rosemont, IL, USA). Following incubation with a horseradish peroxidase-conjugated anti-rabbit antibody, FKBP12 and GAPDH expression were detected on a ChemiDoc MP imaging system (Bio-Rad, Hercules, CA, USA).

### Chromatin immunoprecipitation (ChIP)-qPCR

A total of 10 spores from each *Mucor* strain were inoculated in liquid YPD medium with or without FK506 and incubated overnight at 25°C. The samples were then cross-linked with 1% formaldehyde (ThermoFisher), followed by addition of glycine (Sigma-Aldrich, St. Louis, MO, USA) at a final concentration of 0.125M. After washing and freezing using liquid nitrogen, each sample was ground with a pestle and suspended in ChIP lysis buffer (50 mM HEPES, pH=7.5, 150 mM NaCl, 1 mM EDTA, pH=8, 1% Triton X-100, and 0.1% sodium deoxycholate) containing a protease inhibitor tablet (Roche). Disrupted cells were then sonicated with a Bioruptor (Diagenode) for 40 cycles, with 30s on and 30s off and clarified by centrifuge for 5 min. The input samples were stored at −80°C, while the IP samples were incubated overnight at 4°C with Dynabeads Protein A/G (Invitrogen) pre-conjugated with the anti-histone H3K9 dimethyl antibody (Abcam, Cambridge, UK) or the anti-RNA polymerase II antibody (Active Motif, Carlsbad, CA). Crosslinking between protein and DNA in both input and IP samples was reversed with the MAGnify Chromatin Immunoprecipitation System (Invitrogen), and DNA quality was analyzed using a Qubit (ThermoFisher). qPCR was performed in triplicate with primers described in **S2 Table**. H3K9 methylation enrichment was calculated by normalizing the IP DNA to the input DNA with the ΔCt method and further adjusted to the *actA* gene with the ΔΔCt method, followed by 2^−ΔΔCt^ to determine relative enrichment [57, 58]. RNA polymerase II enrichment was similarly calculated by normalizing the IP DNA to the input DNA (ΔCt), followed by normalization to an intronic region enriched for siRNAs and marked by H3K9me2 (ΔΔCt). Relative enrichment was determined with the 2^−ΔΔCt^ method.

### ChIP sequencing

Input and IP libraries were constructed with KAPA HyperPrep (Roche) and sequenced on a NovaSeq X platform (Illumina) by 150-bp paired end. Raw FASTA files were trimmed with Trimmomatic (v0.39) and filtered reads were mapped to the reference genomes (*M. bainieri* CBS293.63; PRJNA1277536 or *M. atramentarius* CBS202.28; PRJNA1277544) using Bowtie2 (v2.5.4) [59, 60]. The resulting SAM files were converted to BAM files using SAMtools (v1.21) [55]. Broad ChIP-peak calling was analyzed with MACS3 (v3.0.3) with the parameters --mfold 10 1000 and --extsize 147, and enrichment was analyzed by generating coverage files with BamCoverage (v3.5.6), followed by counts per million (CPM) normalization [61, 62]. Processed BAM files were converted to TDF files IGV tools with a window size of 20 bp and the mean window function and subsequently visualized with IGV (v2.16.0) [63]. ChIP-seq experiments were performed with one biological sample per strain.

### *In vivo* infection

Six-week-old male BALB/cAnNCrl mice, 6 to 7 weeks old btained from Charles River laboratory (Wilmington, MA, USA), were analyzed in this study. Five mice were infected with each *Mucor* strain. Two days prior to infection, 200 mg/kg of cyclophosphamide (Sigma-Aldrich), an immunosuppressant, was administered intraperitoneally. Each mouse was infected with 1 × 10^6^ spores (in 50 μL PBS) via retro-orbital injection after anesthetizing the mice with isoflurane for 2.5 min. After infection, the mice were monitored twice daily and administered cyclophosphamide at 200 mg/kg body weight every four days. To assess pathogenicity, weight loss was monitored, and any animal experiencing ≥ 20% loss of body weight was humanely euthanized. All animal experiments in this study were approved by the Duke University Institutional Animal Care and Use Committee (IACUC) (protocol #A224-23-11). Animal care and experiments were conducted according to IACUC ethical guidelines.

### Strains recovered after *in vivo* passage

To assess fungal burden and isolate *Mucor* strains from organs, five mice per *Mucor* strain were intravenously infected. At the humane endpoint, the brain, lung, liver, and spleen were dissected. Whole organs were homogenized in 0.8 mL of PBS with a bead beater for 2 min, then diluted and plated on YPD supplemented with 50μg/mL chloramphenicol. After a two-day incubation at room temperature, fungal colonies were picked and patched onto YPD plates with or without FK506 to assess the stability of FK506 resistance following *in vivo* passage. The stability rate was determined by calculating the proportion of resistant colonies on YPD supplemented with FK506 relative to the total colonies patched on YPD [32].

### Quantification and statistical analyses

Statistical differences between the control and target strains were evaluated using ordinary one-way ANOVA using Tukey’s Honestly Significant Difference (HSD) test with using GraphPad Prism (v10.1.1). Kaplan-Meier survival curves were generated and analyzed with the log-rank test in R (v4.3.2) to estimate survival probability. Data are reported as mean ± standard deviation. In all graphs, p values or asterisks were listed indicating statistical significance (*p < 0.05; **p < 0.01; ***p < 0.001; ****p < 0.0001).

## Figures and Tables

**Fig 1.** FK506-resistant strains isolated from the *Mucor circinelloides* complex. **(A)** Schematic representation of the procedure followed to obtain FK506- and/or rapamycin-resistant strains from *Mucor circinelloides* complex species. This Figure was generated with BioRender.com. **(B)** Phenotypic analysis of FK506-resistant isolates in *M. janssenii* CBS185.68 (PS1), *M. bainieri* CBS293.63 (PS3), and *M. atramentarius* CBS202.28 (PS6). WT, wild-type; M, Mendelian mutant; E, Epimutant.

**Table 1.** FK506 resistant strains isolated in this study.

Species	Name	Phenotype	*fkbA* mutation	Impact on FKBP12	Calcineurin mutation

	M1	FK506-R, Rapa-S	No	No	G1496C in *cnaA* (G347A missense mutation)
	M2	FK506-R, Rapa-R	4,324 bp insertion (Unknown) at 38 bp upstream *fkbA* ORF	-	-
	M3	FK506-R, Rapa-R	Large deletion	Loss	-
	M4	FK506-R, Rapa-R	Large deletion	Loss	-
**PS1 *Mucor janssenii***	M5	FK506-R, Rapa-R	5,052 bp insertion (RC/Helitron_DNA transposons) at 16 bp upstream *fkbA* ORF	-	-
	M6	FK506-R, Rapa-R	C120 1 bp deletion in exon 2 (Frameshift)	Nonsense mutation	-
	M7	FK506-R, Rapa-R	Large deletion	Loss	-
	M8	FK506-R, Rapa-R	G258C in 5’ splice site of intron2	-	-
	M9	FK506-R, Rapa-R	Large deletion	Loss	-
	M10	FK506-R, Rapa-R	G258C in 5’ splice site of intron2	-	-

	E1	FK506-R, Rapa-R	No	No	No
	E2	FK506-R, Rapa-R	No	No	No
	E3	FK506-R, Rapa-R	No	No	No
	E4	FK506-R, Rapa-R	No	No	No
**PS3 *Mucor bainieri***	E5	FK506-R, Rapa-R	No	No	No
	E6	FK506-R, Rapa-R	No	No	No
	M1	FK506-R, Rapa-R	C357 1 bp deletion in exon 3 (Frameshift)	Nonsense mutation	No
	E7	FK506-R, Rapa-R	No	No	No
	E8	FK506-R, Rapa-R	No	No	No
	E9	FK506-R, Rapa-R	No	No	No

	M1	FK506-R, Rapa-R	C404 1 bp deletion in exon 3 (Frameshift)	Nonsense mutation	No
	M2	FK506-R, Rapa-S	No	No	G477T in *cnbR* (V122F missense mutation)
	M3	FK506-R, Rapa-R	G249A in 5’ splice site	No	No
	M4	FK506-R, Rapa-R	G249A in 5’ splice site	No	No
**PS6 *Mucor atramentarius***	M5	FK506-R, Rapa-S	G189T in exon 2	Synonymous mutation	No
	M6	FK506-R, Rapa-R	C404 1 bp deletion in exon 3 (Frameshift)	Nonsense mutation	No
	M7	FK506-R, Rapa-R	T2C in exon 1 (start-loss mutation)	Loss	No
	E1	FK506-R, Rapa-R	No	No	No
	E2	FK506-R, Rapa-R	No	No	No
	M8	FK506-R, Rapa-R	C404 1 bp deletion in exon 3 (Frameshift)	Nonsense mutation	No

**Table 2.** Epimutant reverted strains analyzed in this study.

Species	Name	Background	Phenotype	Description

	E1P4	E1	FK506-S, Rapa-S	E1 Revertant after passage 4
	E2P4	E2	FK506-S, Rapa-S	E2 Revertant after passage 4
	E3P12	E3	FK506-S, Rapa-S	E3 Revertant after passage 12
**PS3 *Mucor bainieri***	E3P16	E4	FK506-S, Rapa-S	E4 Revertant after passage 16
	E5P12	E5	FK506-S, Rapa-S	E5 Revertant after passage 12
	E6P12	E6	FK506-S, Rapa-S	E6 Revertant after passage 12
	E7P28	E7	FK506-S, Rapa-S	E7 Revertant after passage 28
	E8P28	E8	FK506-S, Rapa-S	E8 Revertant after passage 28
	E9P24	E9	FK506-S, Rapa-S	E9 Revertant after passage 24

**PS6 *Mucor atramentarius***	E1P32	E1	FK506-S, Rapa-S	E1 Revertant after passage 32
	E2P28	E2	FK506-S, Rapa-S	E2 Revertant after passage 28

**Table 3.** Summary of epigenetic mechanisms in *Mucor bainieri*.

Group	Mechanisms	Epimutant	Revertant	Average passages for revertant

1	RNAi-associated heterochromatin-dependent silencing	E1	E1P4	
		E2	E2P4	6.67
		E3	E3P12	

2	Heterochromatin-dependent silencing	E4	E3P16	16

3	Canonical RNAi-dependent silencing	E5	E5P12	18.8
		E6	E6P12	
		E7	E7P28	
		E8	E8P28	
		E9	E9P24	

## Data Availability

Small RNA sequencing and ChIP sequencing raw data are available in the NCBI SRA under the project accession numbers PRJNA1242486 (*M. bainieri* CBS293.63) and PRJNA1242487 (*M. atramentarius* CBS202.28).
