# Supplementary material for: Epimutations driven by RNAi or heterochromatin evoke transient antimicrobial drug resistance in fungi
Source: bioRxiv. 2025 Nov 25:2025.06.17.660219. Preprint. [Version 4] doi: 10.1101/2025.06.17.660219 (PMC12236829; doi:10.1101/2025.06.17.660219)
Supplement: Supplement 1 [file media-1.pdf]

Figure S1. Son et al.

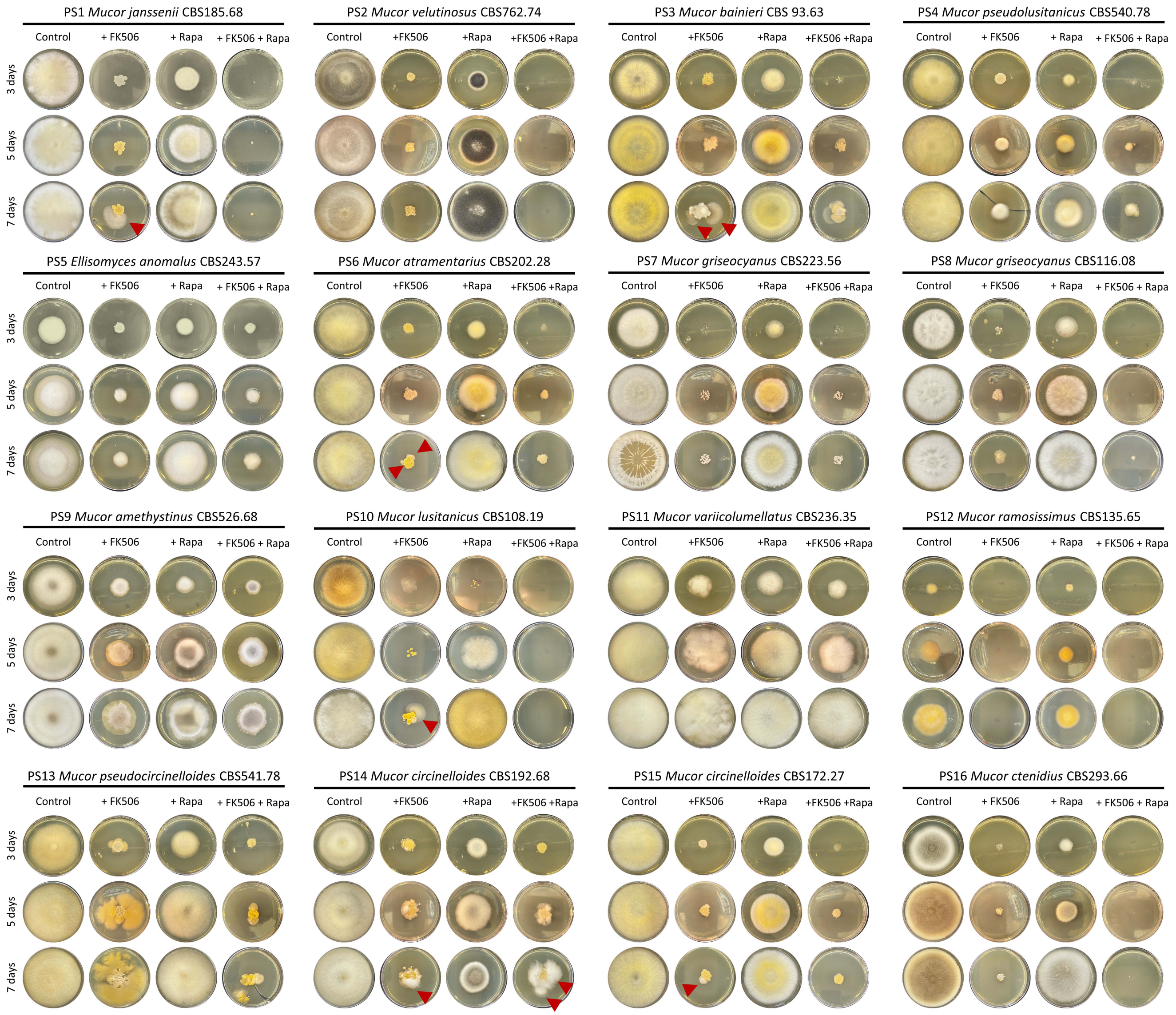

Figure S2. Son et al.

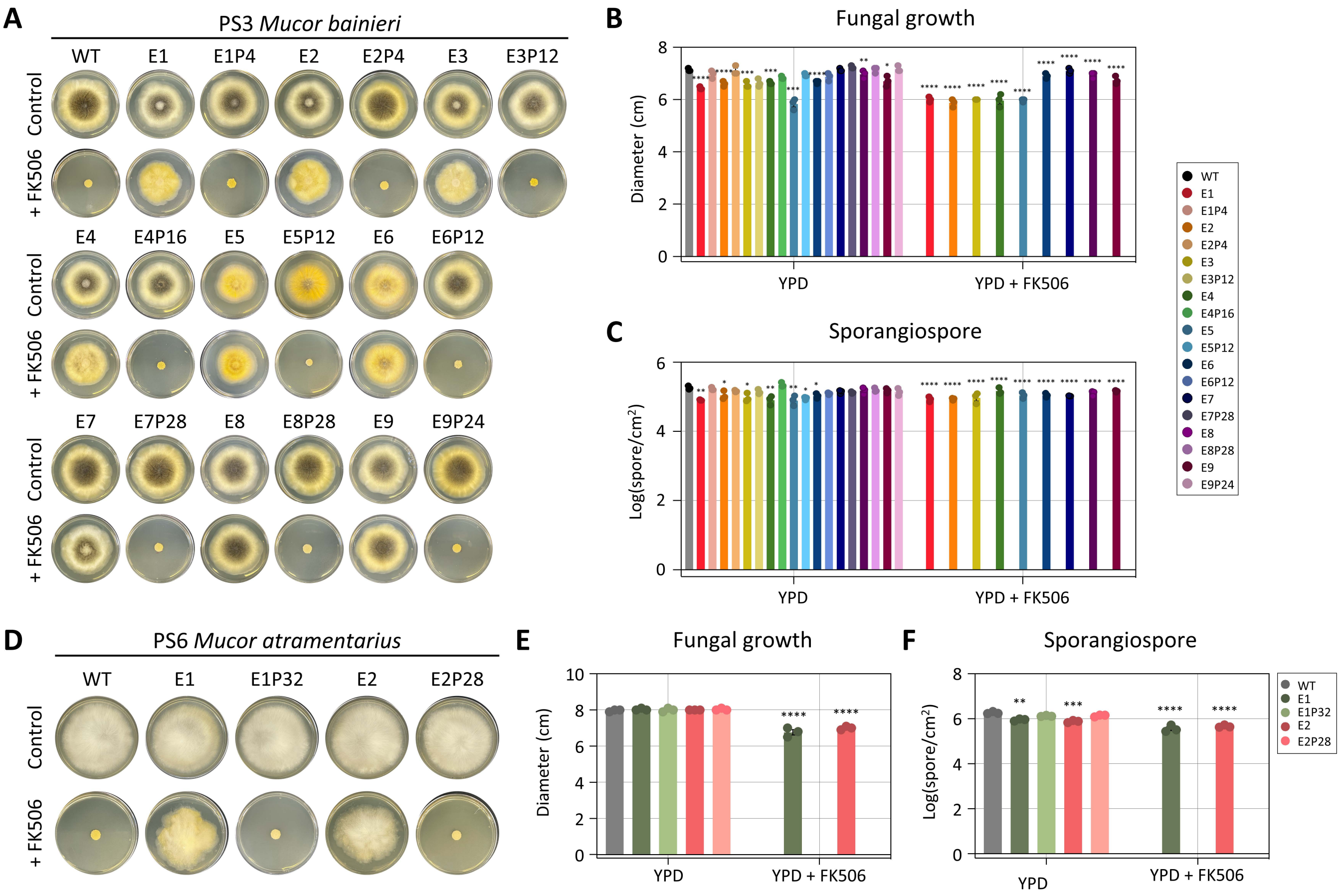

Figure S3. Son et al.

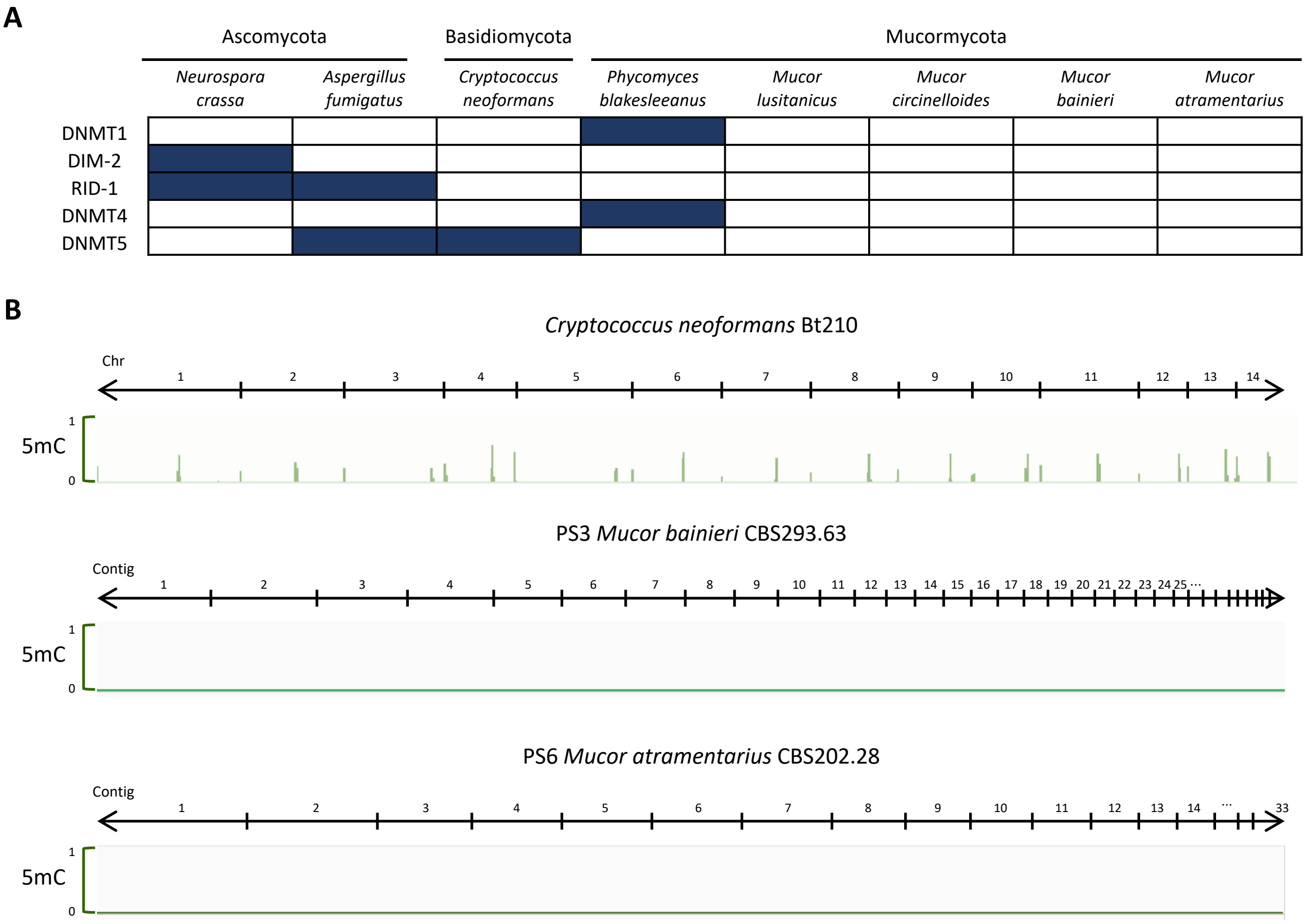

Figure S4. Son et al.

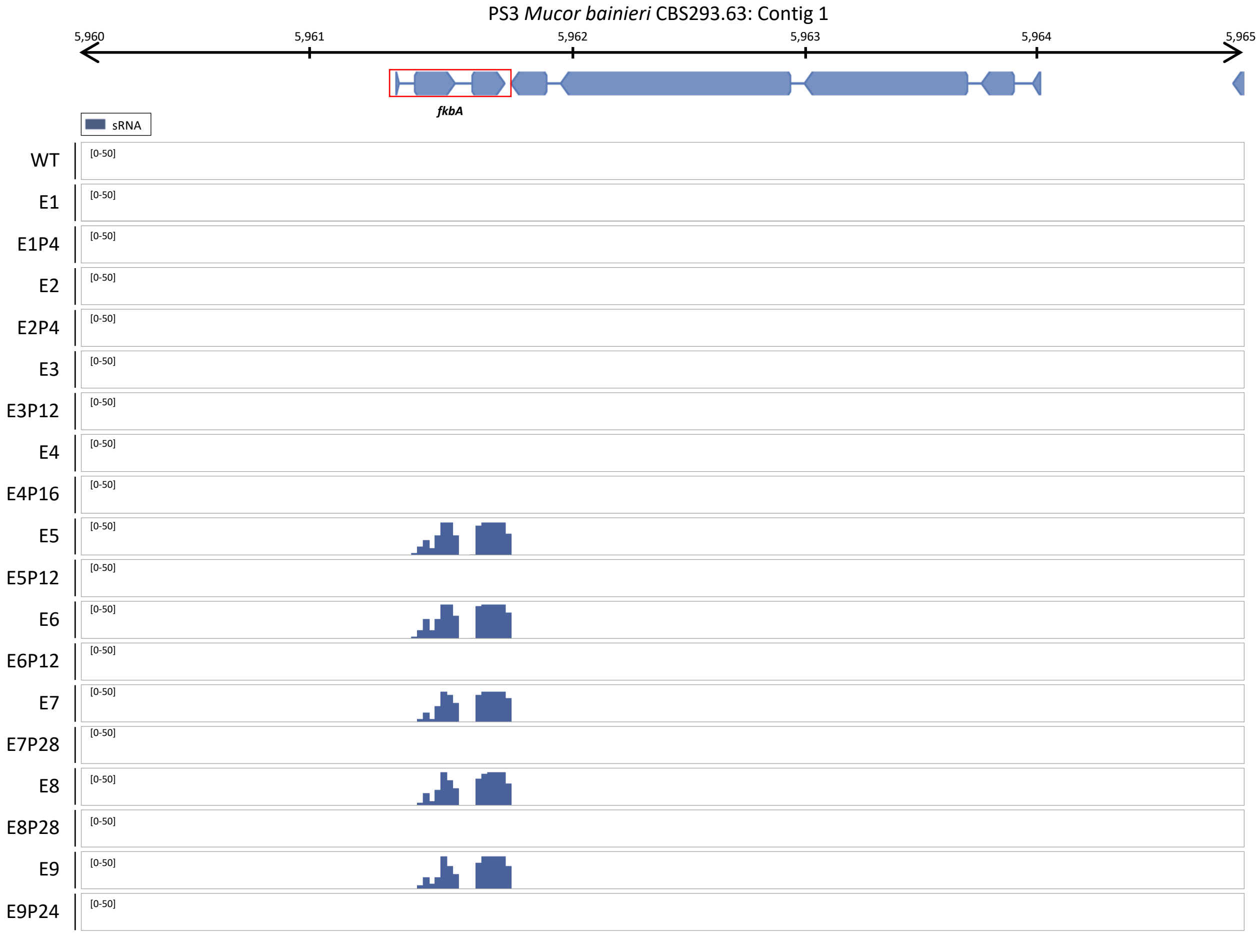

Figure S5. Son et al.

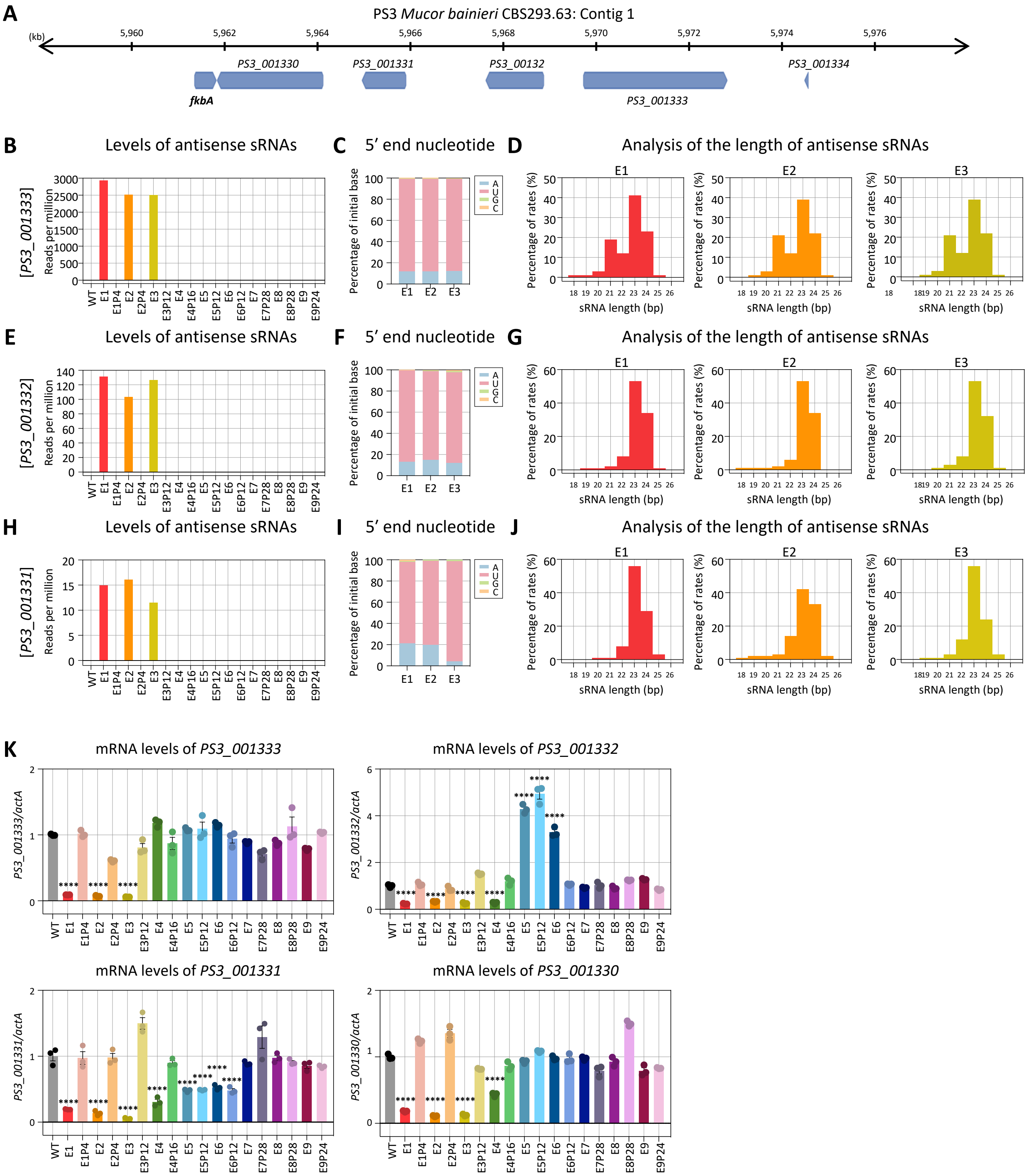

Figure S6. Son et al.

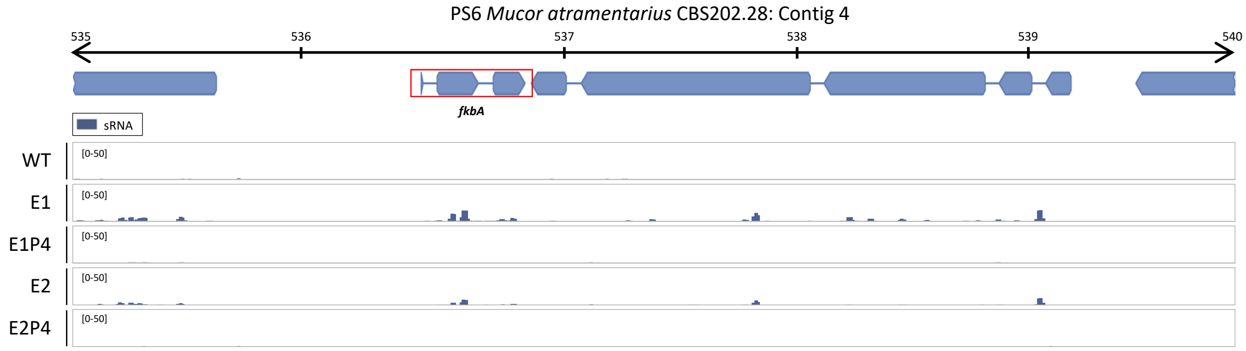

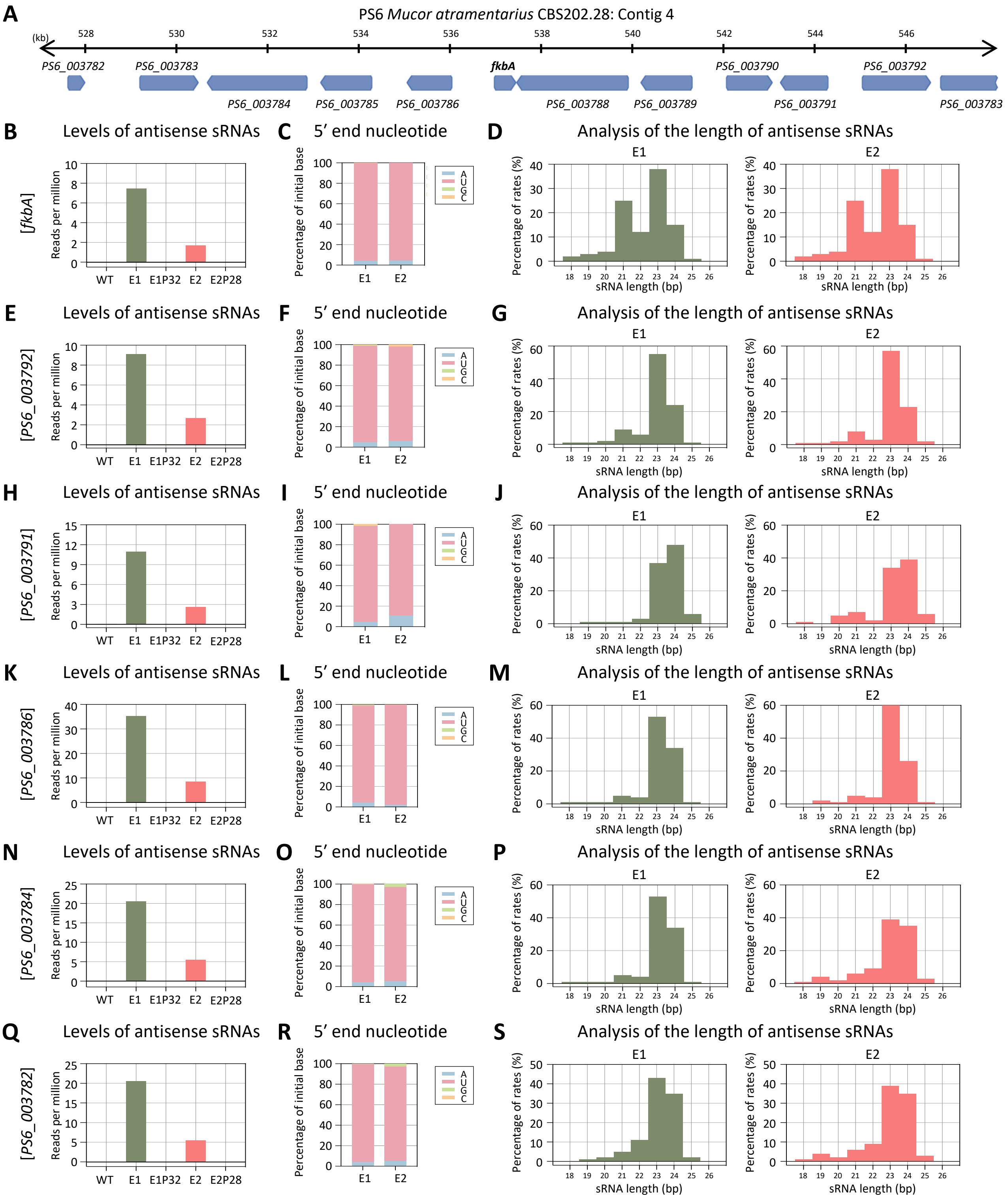
A
PS6 Mucor atramentarius CBS202.28: Contig 4
(kb)
528
530
532
534
536
538
540
542
544
546
PS6_003782
PS6_003783
PS6_003784
PS6_003785
PS6_003786
fkbA
PS6_003788
PS6_003789
PS6_003790
PS6_003791
PS6_003792
PS6_003783
B Levels of antisense sRNAs
C 5' end nucleotide
D Analysis of the length of antisense sRNAs
[fkbA]
E Levels of antisense sRNAs
F 5' end nucleotide
G Analysis of the length of antisense sRNAs
[PS6_003792]
H Levels of antisense sRNAs
I 5' end nucleotide
J Analysis of the length of antisense sRNAs
[PS6_003791]
K Levels of antisense sRNAs
L 5' end nucleotide
M Analysis of the length of antisense sRNAs
[PS6_003786]
N Levels of antisense sRNAs
O 5' end nucleotide
P Analysis of the length of antisense sRNAs
[PS6_003784]
Q Levels of antisense sRNAs
R 5' end nucleotide
S Analysis of the length of antisense sRNAs
[PS6_003782]
Reads per million
WT
E1
E1P32
E2
E2P28
Percentage of initial base
A
U
G
C
E1
E2
Percentage of rates (%)
sRNA length (bp)
18
19
20
21
22
23
24
25
26

Figure S8. Son et al.

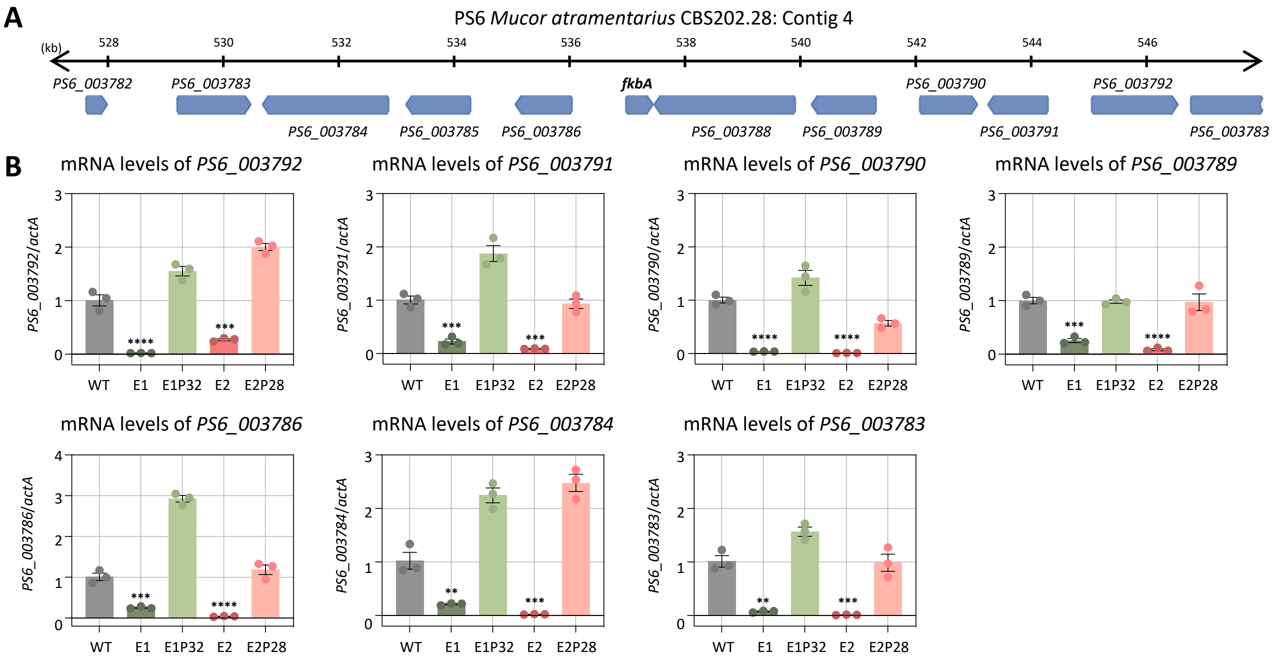

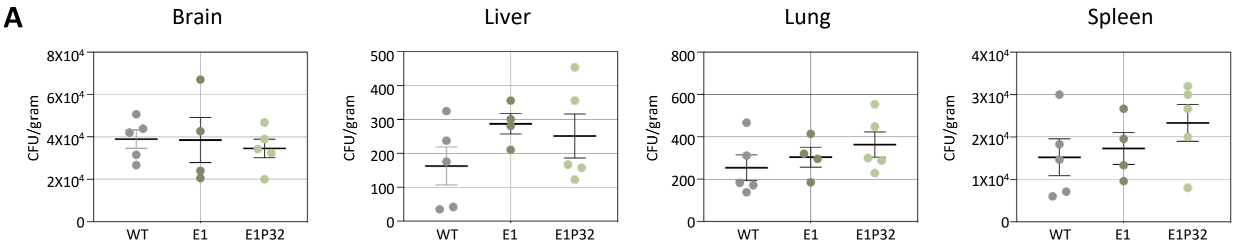
A
Brain
Liver
Lung
Spleen
CFU/gram
8X10^4
6X10^4
4X10^4
2X10^4
0
500
400
300
200
100
800
600
3X10^4
1X10^4
WT
E1
E1P32

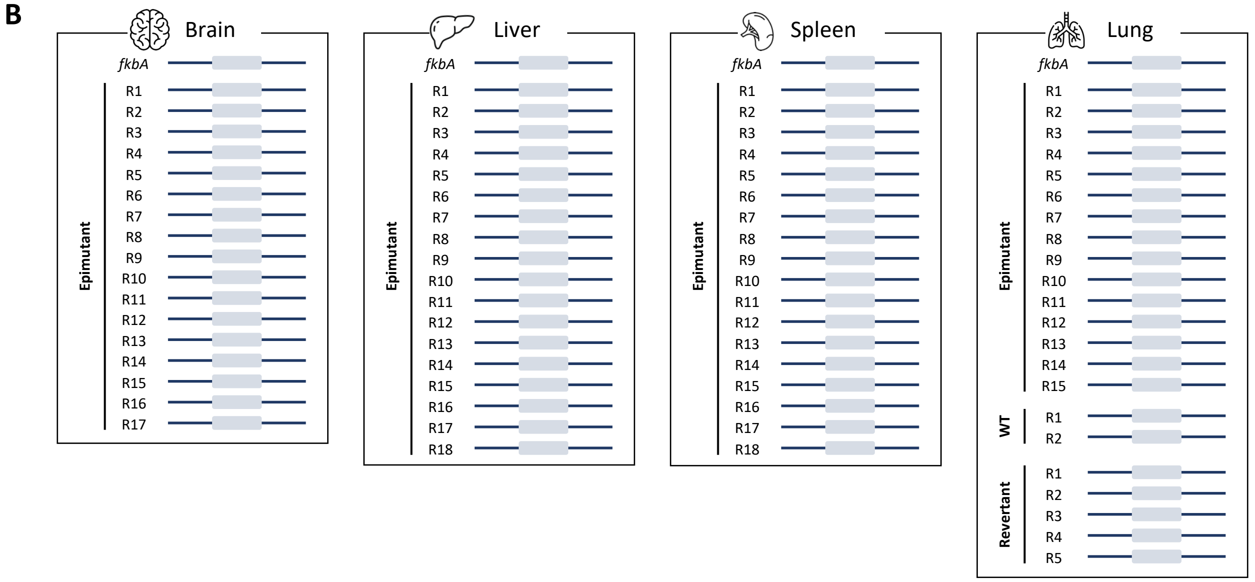
B
Brain
Liver
Spleen
Lung
fkbA
Epimutant
WT
Revertant
R1
R2
R3
R4
R5
R6
R7
R8
R9
R10
R11
R12
R13
R14
R15
R16
R17
R18

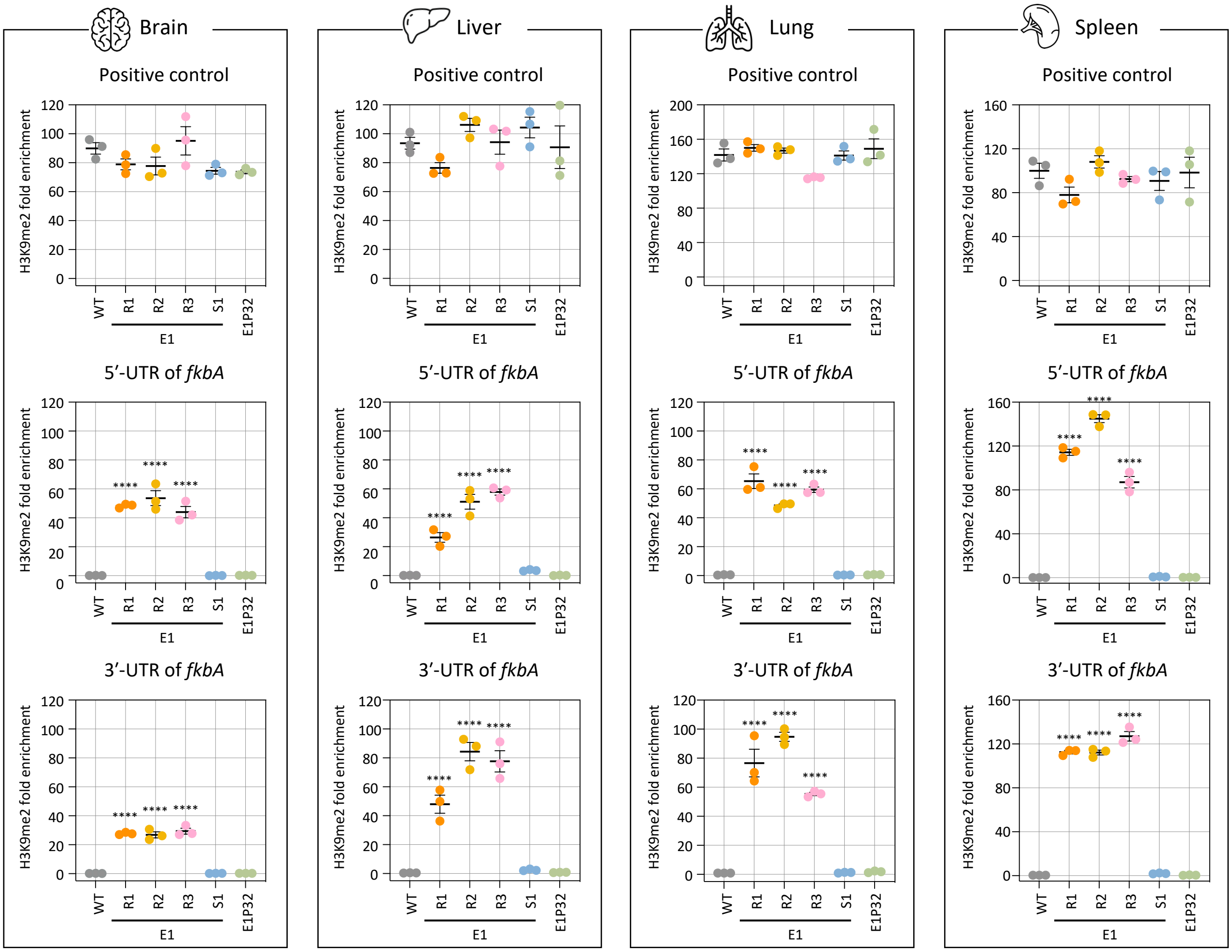
Brain
Liver
Lung
Spleen
Positive control
5′-UTR of fkbA
3′-UTR of fkbA
H3K9me2 fold enrichment
WT
R1
R2
R3
S1
E1P32
E1
****

Figure S11. Son et al.

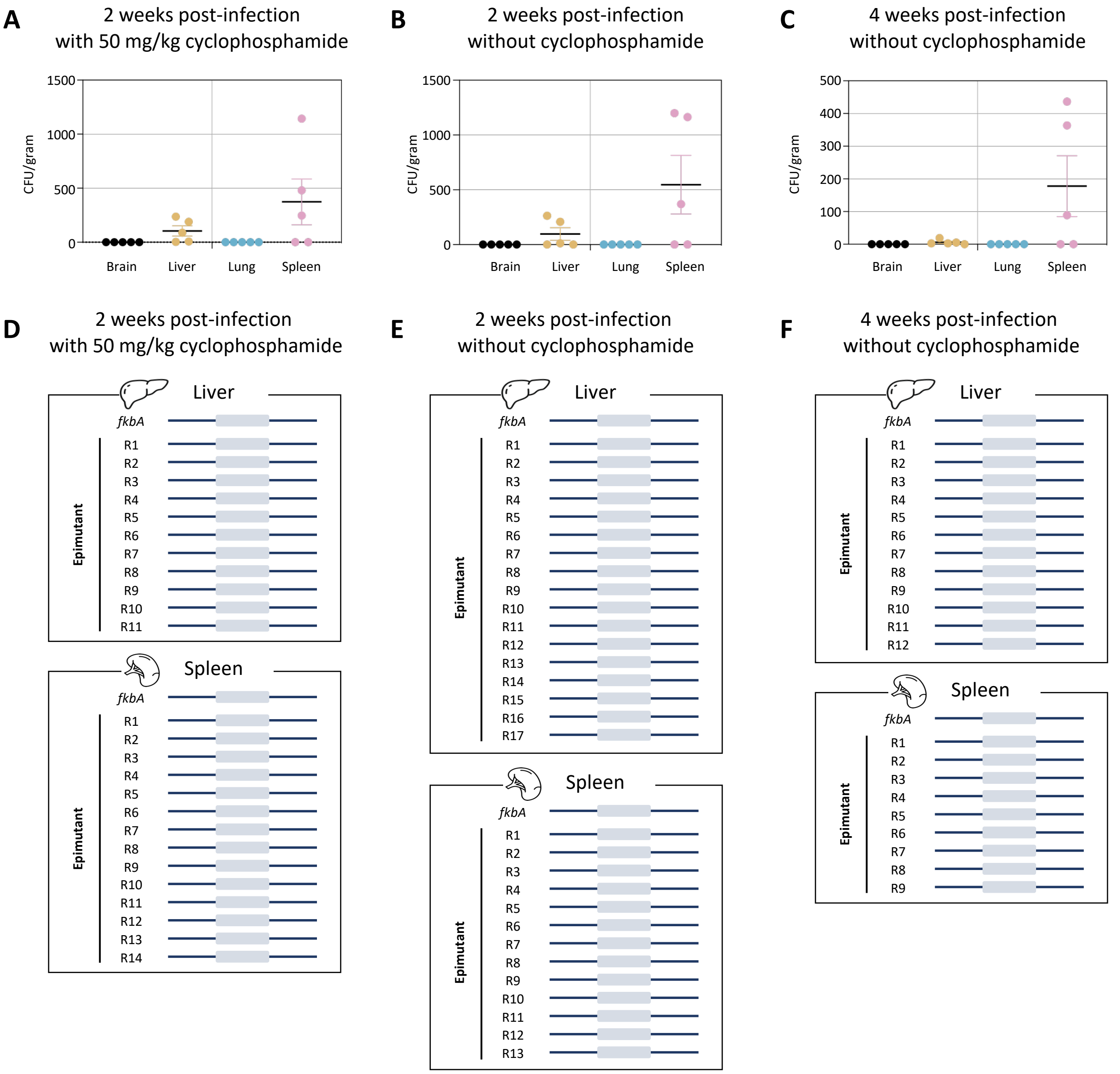

Figure S12. Son et al.

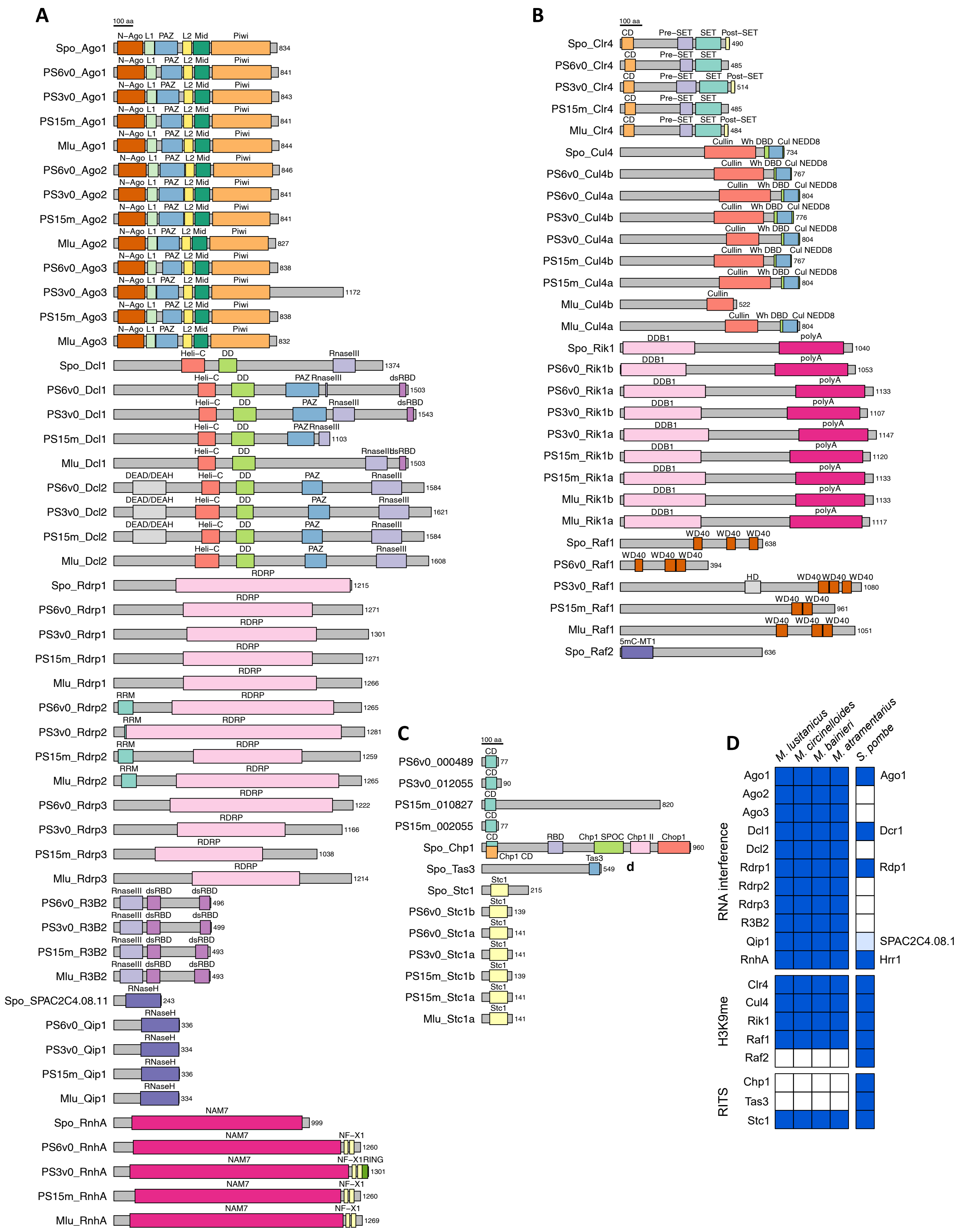

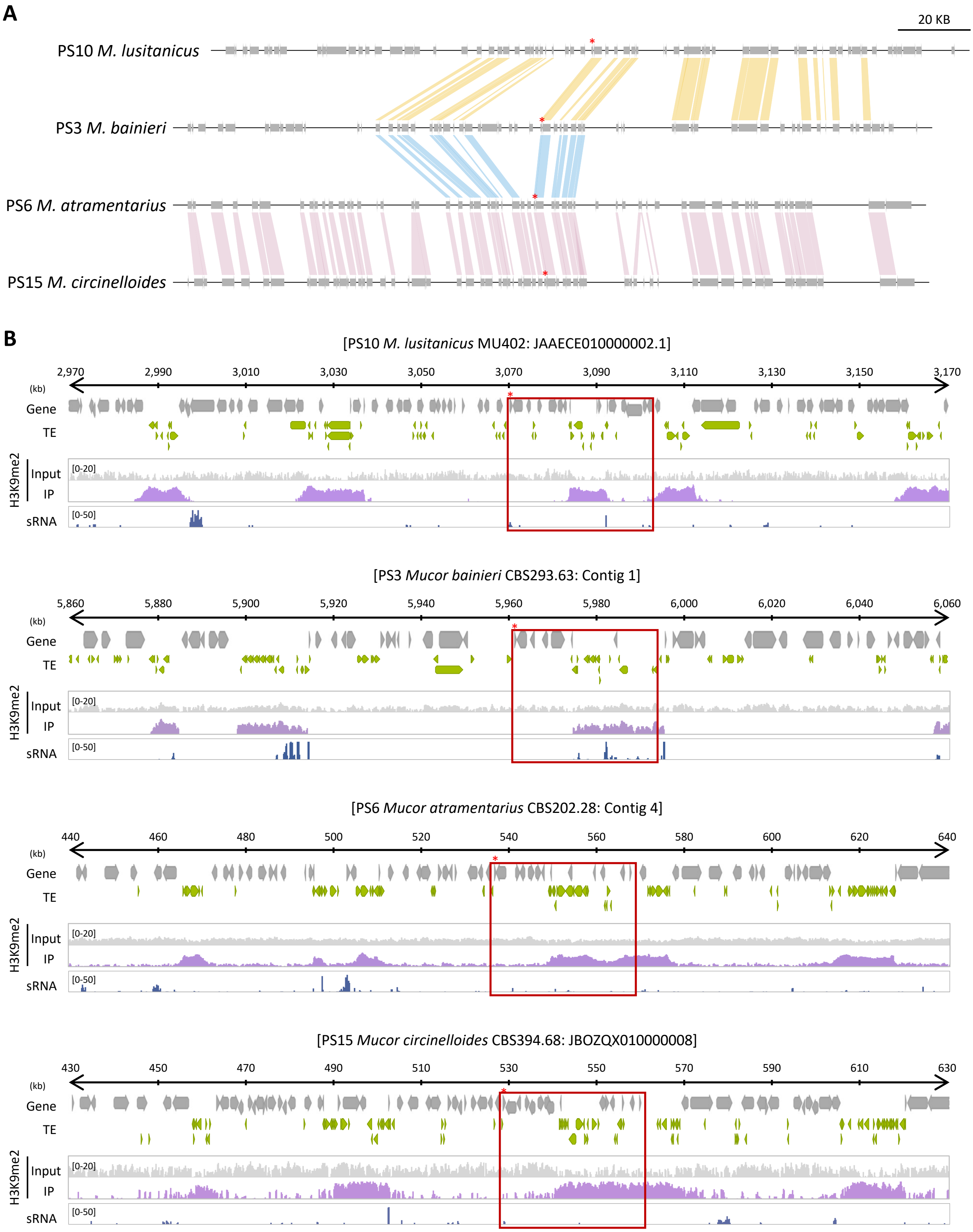
A
20 KB
PS10 M. lusitanicus
PS3 M. bainieri
PS6 M. atramentarius
PS15 M. circinelloides
B
[PS10 M. lusitanicus MU402: JAAECE010000002.1]
2,970 2,990 3,010 3,030 3,050 3,070 3,090 3,110 3,130 3,150 3,170
(kb)
Gene
TE
H3K9me2
Input
IP
[0-20]
sRNA
[0-50]
[PS3 Mucor bainieri CBS293.63: Contig 1]
5,860 5,880 5,900 5,920 5,940 5,960 5,980 6,000 6,020 6,040 6,060
[PS6 Mucor atramentarius CBS202.28: Contig 4]
440 460 480 500 520 540 560 580 600 620 640
[PS15 Mucor circinelloides CBS394.68: JBOZQX010000008]
430 450 470 490 510 530 550 570 590 610 630

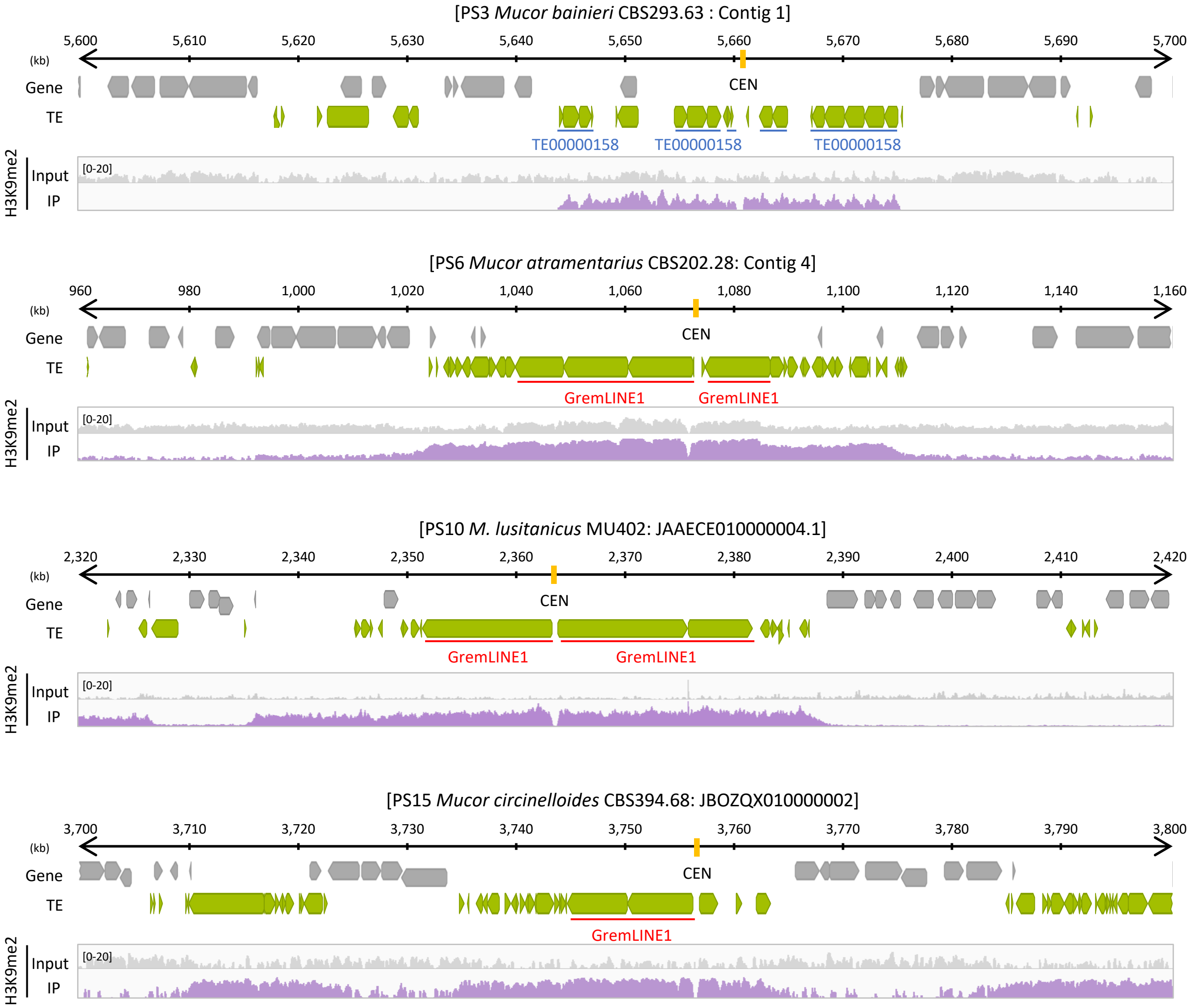
[PS3 Mucor bainieri CBS293.63 : Contig 1]
5,600
5,610
5,620
5,630
5,640
5,650
5,660
5,670
5,680
5,690
5,700
(kb)
Gene
TE
CEN
TE00000158
TE00000158
TE00000158
H3K9me2
Input
IP
[0-20]
[PS6 Mucor atramentarius CBS202.28: Contig 4]
960
980
1,000
1,020
1,040
1,060
1,080
1,100
1,120
1,140
1,160
(kb)
Gene
TE
CEN
GremLINE1
GremLINE1
H3K9me2
Input
IP
[0-20]
[PS10 M. lusitanicus MU402: JAAECE010000004.1]
2,320
2,330
2,340
2,350
2,360
2,370
2,380
2,390
2,400
2,410
2,420
(kb)
Gene
TE
CEN
GremLINE1
GremLINE1
H3K9me2
Input
IP
[0-20]
[PS15 Mucor circinelloides CBS394.68: JBOZQX010000002]
3,700
3,710
3,720
3,730
3,740
3,750
3,760
3,770
3,780
3,790
3,800
(kb)
Gene
TE
CEN
GremLINE1
H3K9me2
Input
IP
[0-20]
