# Supplementary material for: Epimutations driven by RNAi or heterochromatin evoke antifungal drug resistance in human fungal pathogens
Source: bioRxiv. 2025 Jul 11:2025.06.17.660219. Preprint. [Version 3] doi: 10.1101/2025.06.17.660219 (PMC12236829; doi:10.1101/2025.06.17.660219)
Supplement: Supplementary file 1 [file media-1.zip › Supplementary Figure.pdf]

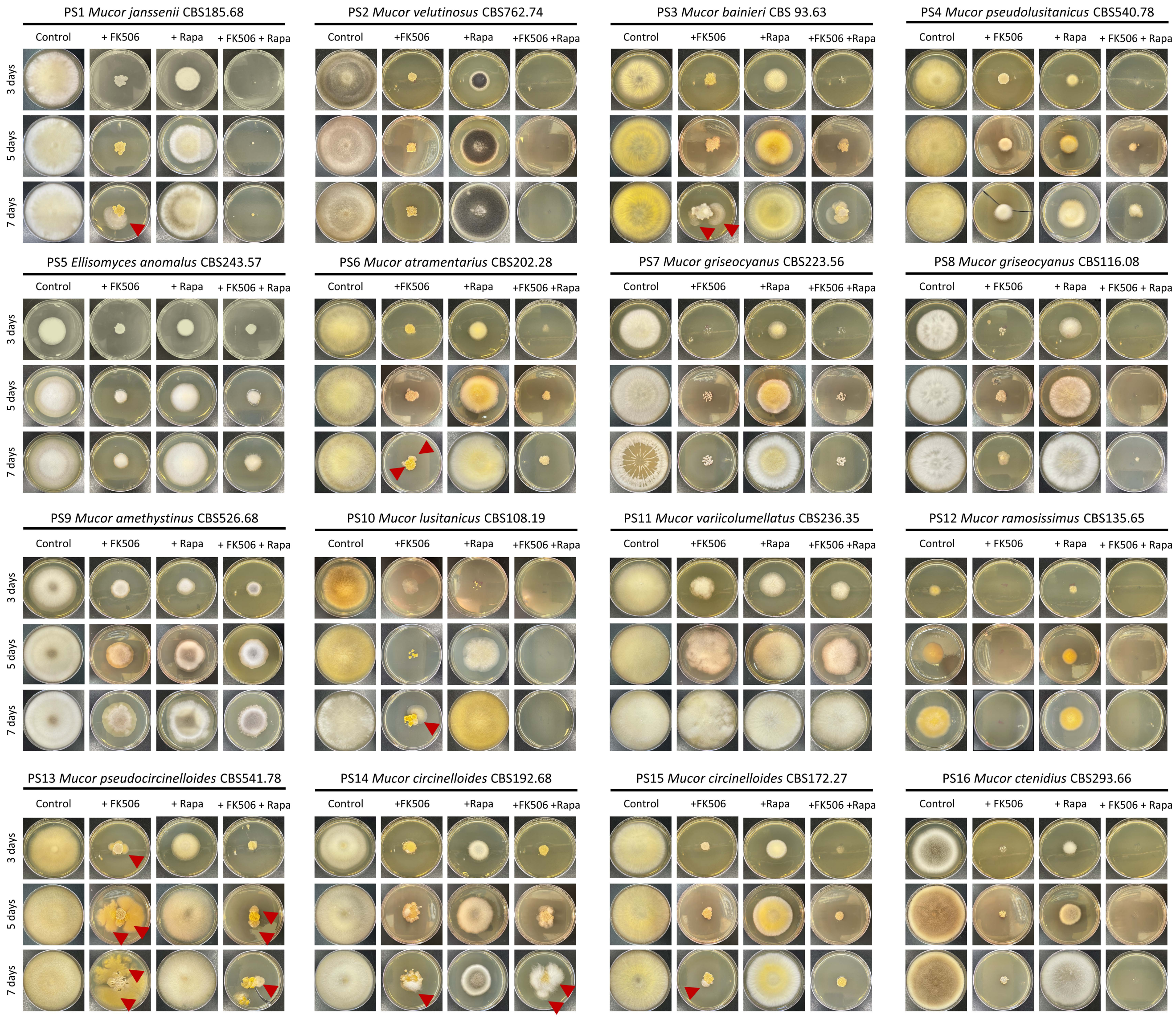

Figure S1. Isolation of FK506- and rapamycin-resistant strains from the *Mucor circinelloides* complex.

Spores from phylogenetic species (PS) 1 to PS16 were point-inoculated onto YPD plates and incubated at room temperature until resistant colonies emerged. After seven days, resistant colonies were observed in PS1, PS3, PS6, PS10, PS13, PS14, and PS15. PS10, PS14, and PS15 were included as positive controls, as resistant strains had previously been characterized. Red arrows indicate sectors of FK506-resistant growth on the plate. FK506 was used at 1 μg/mL and rapamycin at 100 ng/mL.

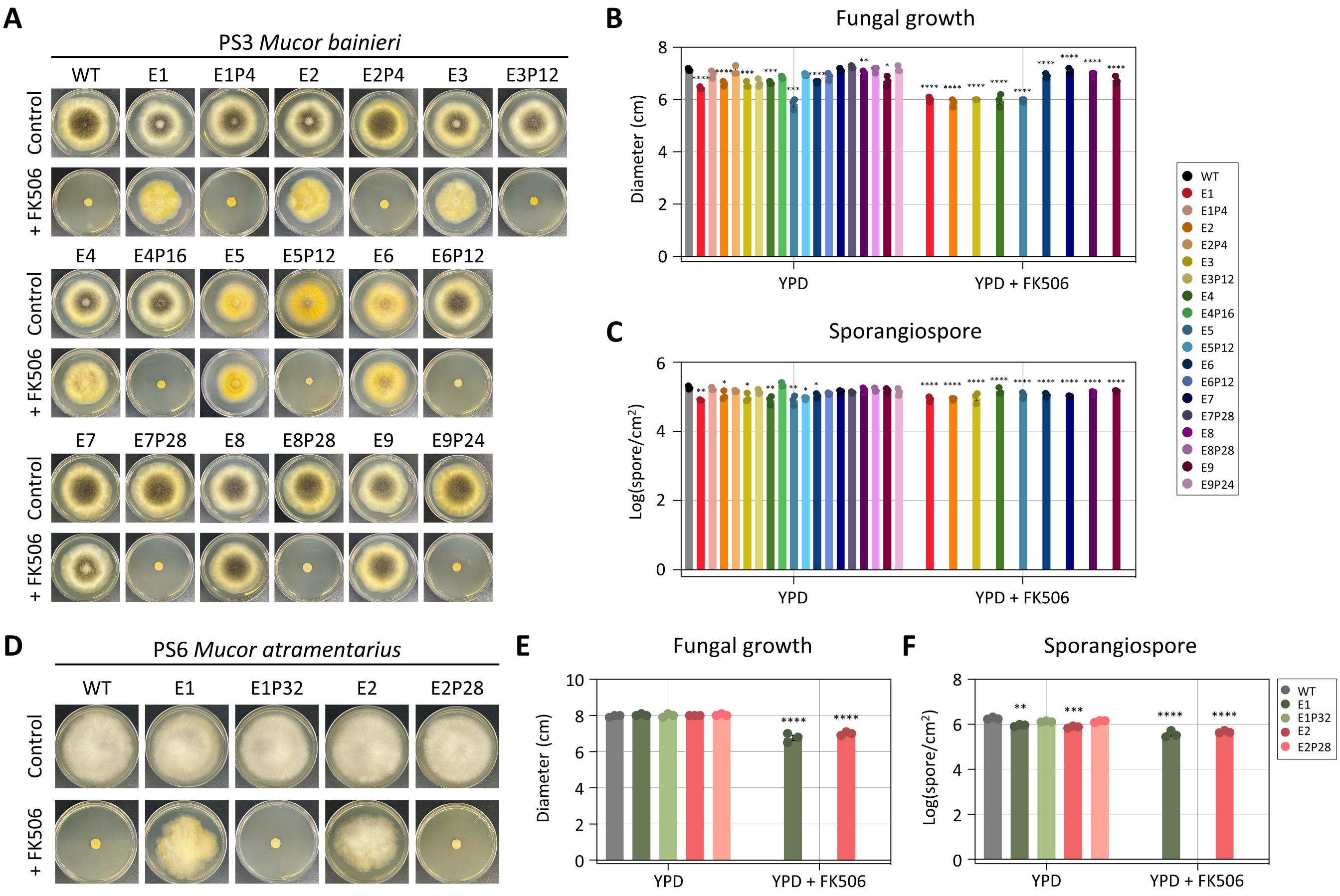

Figure S2. Fungal growth and sporangiospore production in *M. bainieri* and *M. atramentarius.*

(A, D) Point-inoculation phenotypes of *Mucor* strains grown on YPD with or without 1 μg/mL FK506 for 4 days.

(B, E) Quantification of fungal growth for the same strains shown in (A or D). Error bars represent mean ± SEM (n = 3). Statistical significance: $^{*}p \leq 0.05$; $^{**}p \leq 0.01$; $^{***}p \leq 0.001$; $^{****}p \leq 0.0001$.

(C, F) Quantification of sporangiospore production for the same strains shown in (A or D). Error bars represent mean ± SEM (n = 3). Statistical significance: ; $^{**}p \leq 0.01$; $^{***}p \leq 0.001$; $^{****}p \leq 0.0001$.

WT, wild-type; E1–E9, epimutants; E1P4–E9P24, revertants.

Figure S3. Son et al.

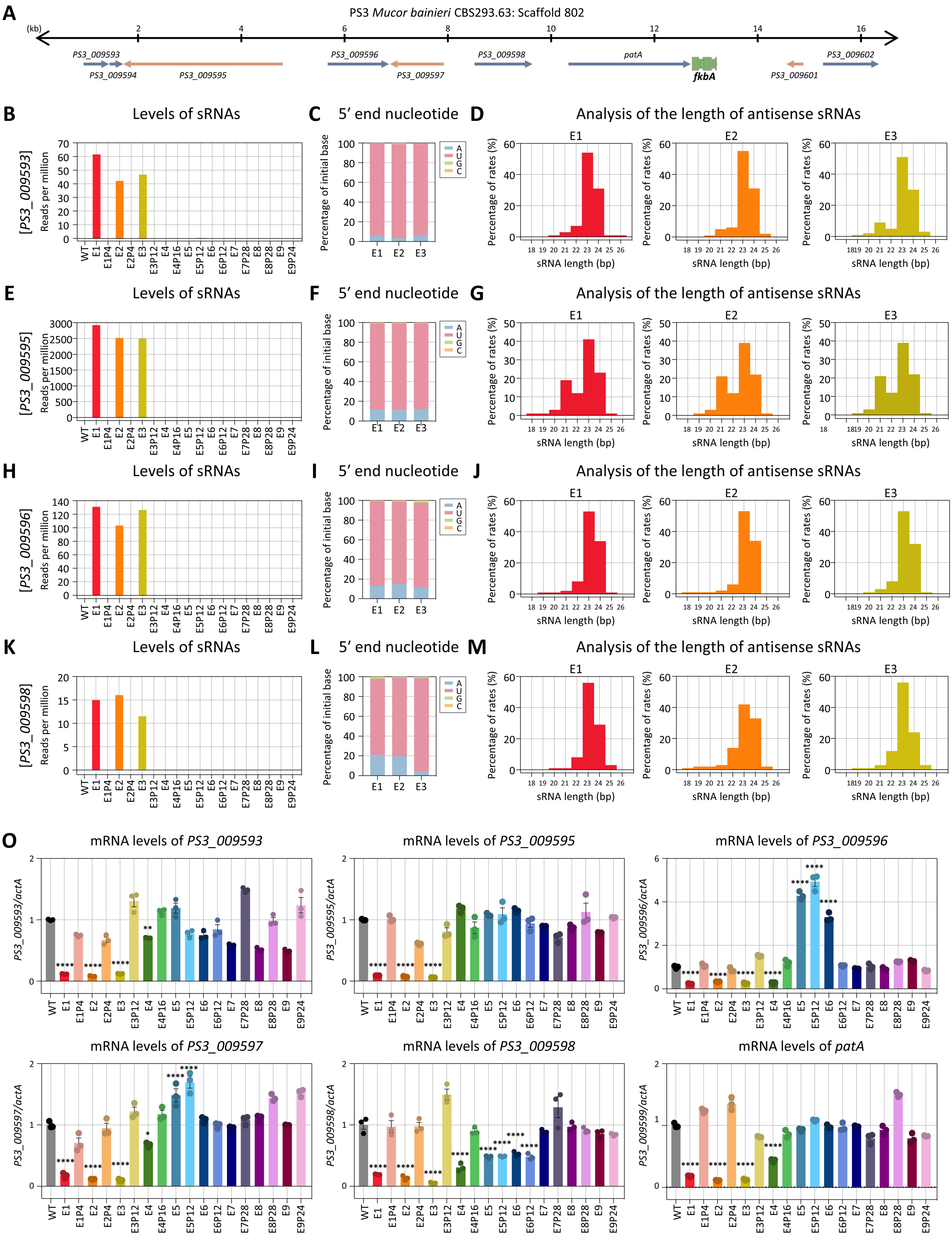

Figure S3. Profiling of small RNAs and relative mRNA abundance for genes neighboring *fkbA* in *M. bainieri*.
(A) Genomic schematic of the *fkbA* locus and its flanking genes.
(B, E, H, K) Small RNA abundance (RPM: reads per million) mapped to genes adjacent to *fkbA*.
(C, F, I, L) 5′-end nucleotide preference of small RNAs associated with each *fkbA*-neighboring gene.
(D, G, J, M) Size distribution of small RNAs mapped to each *fkbA*-neighboring gene.
(O) Quantification of mRNA expression in genes adjacent to *fkbA*. Error bars represent mean ± SEM (n = 3).
Statistical significance: $^{*}p \le 0.05$; $^{**}p \le 0.01$; $^{****}p \le 0.0001$.
WT, wild-type; E1–E9, epimutants; E1P4–E9P24, revertants.

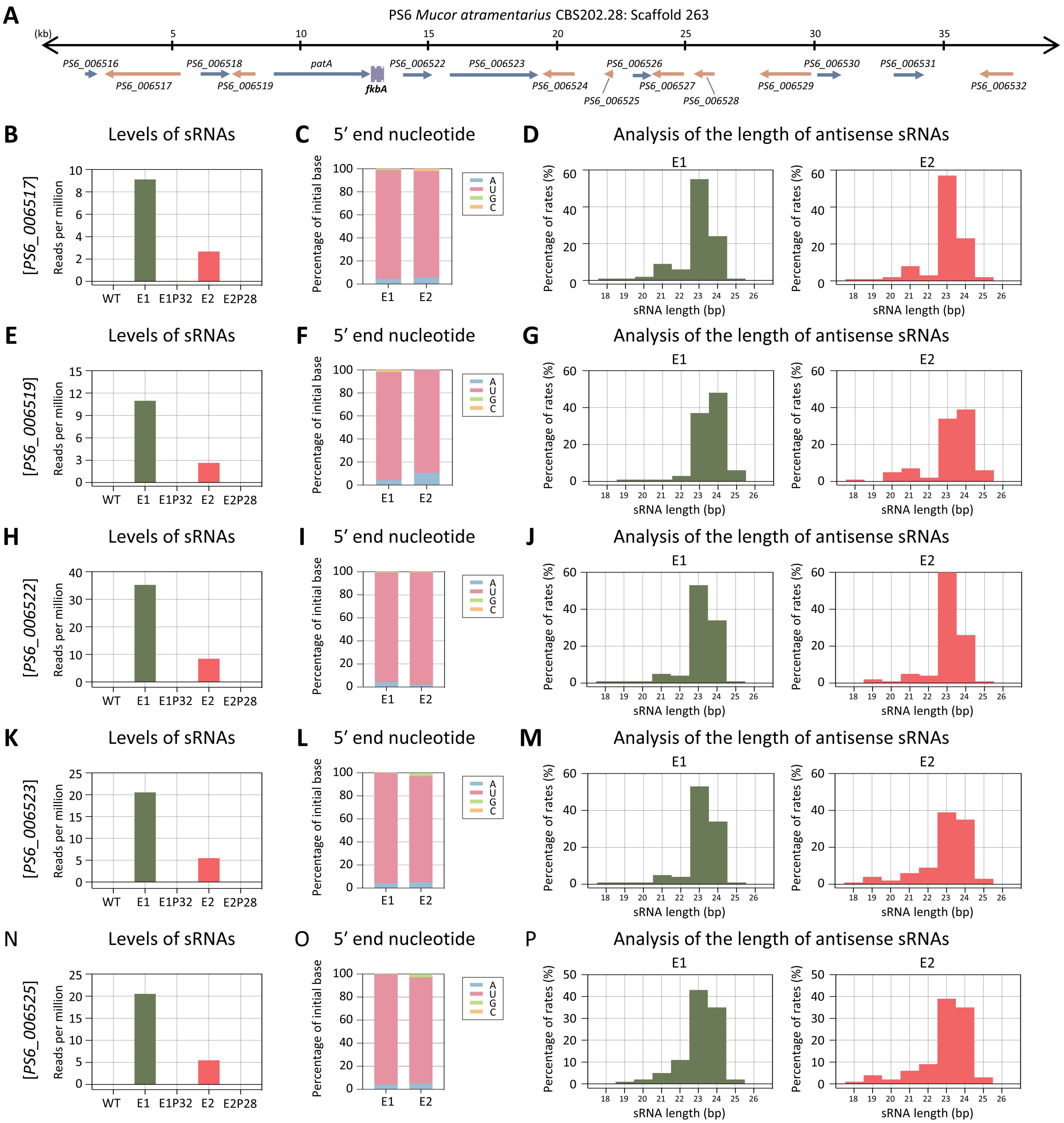

Figure S4. Identification and profiling of small RNAs in genes neighboring *fkbA* in *M. atramentarius*.
(A) Genomic schematic of the *fkbA* locus and its flanking genes.
(B, E, H, K, N) Small RNA abundance (RPM: reads per million) mapped to genes adjacent to *fkbA*.
(C, F, I, L, O) 5′-end nucleotide preference of small RNAs associated with each *fkbA*-neighboring gene.
(D, G, J, M, P) Size distribution of small RNAs mapped to each *fkbA*-neighboring gene.
WT, wild-type; E1 and E2, epimutants; E1P32 and E2P28, revertants.

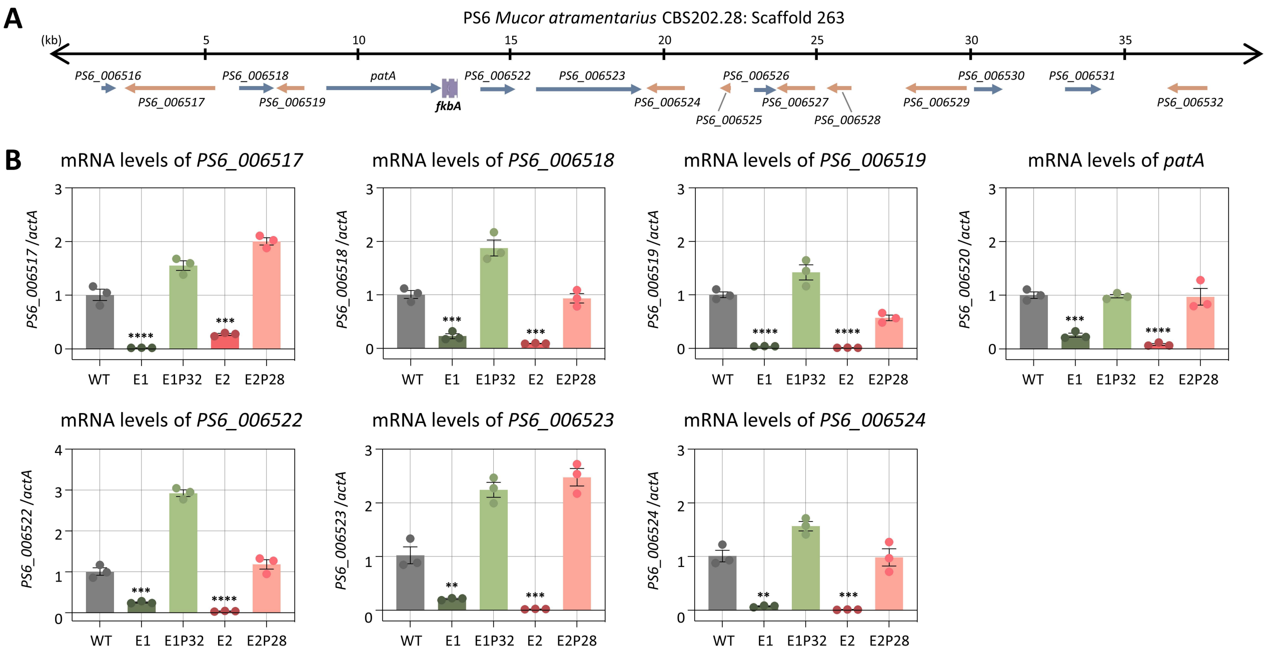

Figure S5. Relative mRNA abundance of genes neighboring *fkbA* in *M. atramentarius*.

(A) Genomic schematic of the *fkbA* locus and its flanking genes.

(B) Quantification of mRNA expression in genes adjacent to *fkbA*. Error bars represent mean ± SEM (n = 3). Statistical significance: **$p \leq 0.01$, ***$p \leq 0.001$ and ****$p \leq 0.0001$. WT, wild-type; E1 and E2, epimutants; E1P32 and E2P28, revertants.

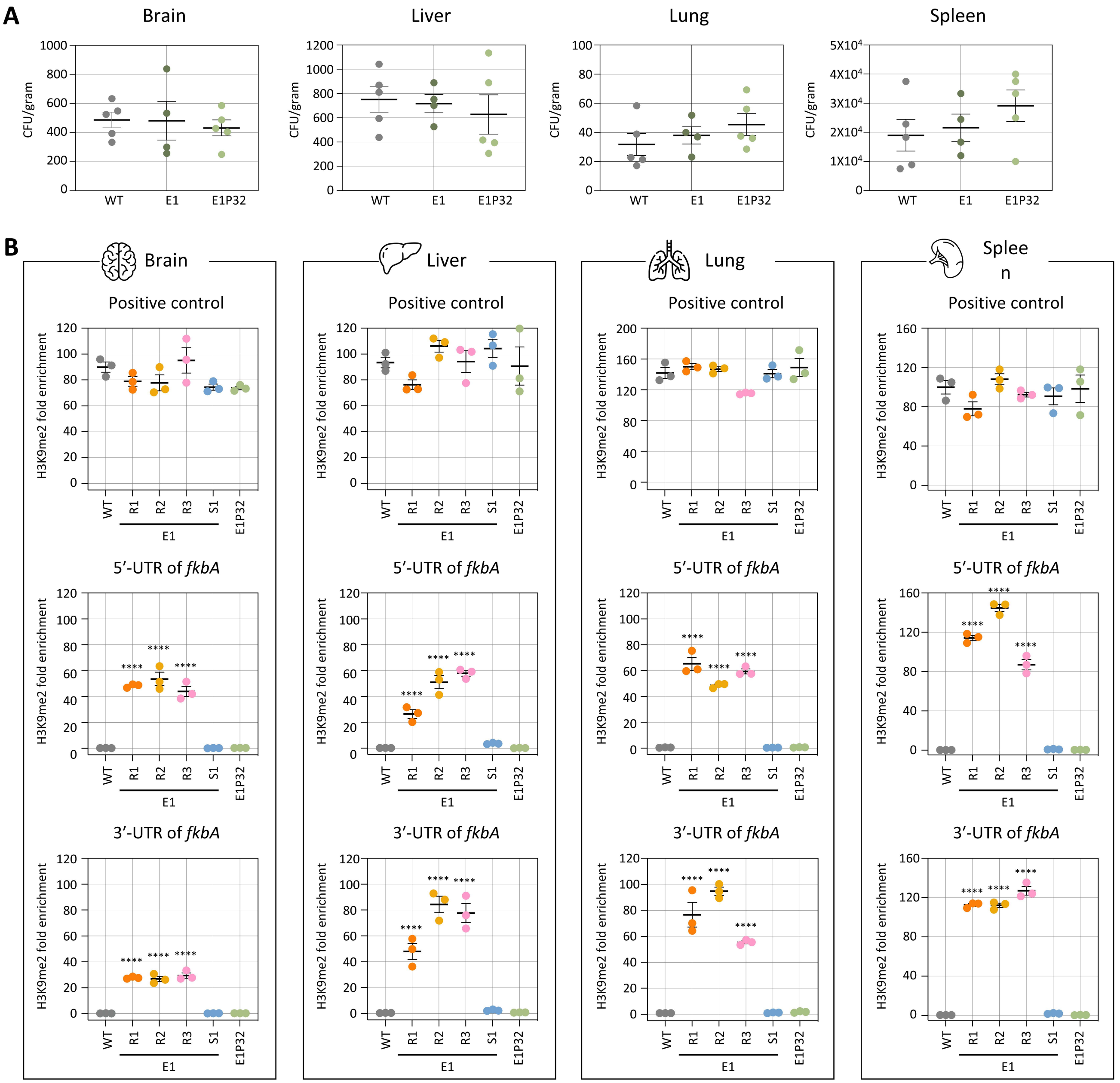

Figure S6. ***In vivo*** **fungal burden and chromatin profiling at the** ***fkbA*** **locus in** ***M. atramentarius*****.**

(A) Fungal burden following retro-orbital inoculation of *M. atramentarius*, expressed as colony-forming units (CFU) per gram of tissue from four organs. Five biological replicates were assessed. Error bars represent mean ± SEM (n = 3). WT, wild-type; E1, epimutant; E1P32, revertant.

(B) ChIP-qPCR analysis of H3K9me2 enrichment at the *fkbA* locus following *in vivo* passage, targeting the positive control region, 5′-UTR, and 3′-UTR. Error bars represent mean ± SEM (n = 3). Statistical significance: ***$p \leq 0.001$; ****$p \leq 0.0001$. WT, wild-type; E1R1–E1R3, FK506-resistant epimutants; E1S1, FK506-sensitive epimutant; E1P32, revertant.
