# Supplementary material for: Epimutations driven by RNAi or heterochromatin evoke antifungal drug resistance in human fungal pathogens
Source: bioRxiv. 2025 Jul 11:2025.06.17.660219. Preprint. [Version 3] doi: 10.1101/2025.06.17.660219 (PMC12236829; doi:10.1101/2025.06.17.660219)
Supplement: Supplementary file 2 [file media-2.zip › Supplementary table.pdf]

Table S1. *Mucor* strains analyzed in this study.

Species	Strain name	Description	References
Mucor janssenii	CBS 185.68	Phylogenetic species 1	[1]
Mucor janssenii	YES1	Mutant 1 (M1)	This study
Mucor janssenii	YES2	Mutant 2 (M2)	This study
Mucor janssenii	YES3	Mutant 3 (M3)	This study
Mucor janssenii	YES4	Mutant 4 (M4)	This study
Mucor janssenii	YES5	Mutant 5 (M5)	This study
Mucor janssenii	YES6	Mutant 6 (M6)	This study
Mucor janssenii	YES7	Mutant 7 (M7)	This study
Mucor janssenii	YES8	Mutant 8 (M8)	This study
Mucor janssenii	YES9	Mutant 9 (M9)	This study
Mucor janssenii	YES10	Mutant 10 (M10)	This study
Mucor velutinosus	CBS 762.74	Phylogenetic species 2	[1]
Mucor bainieri	CBS 293.63	Phylogenetic species 3	[1]
Mucor bainieri	YES12	Epimutant 1 (E1)	This study
Mucor bainieri	YES13	Epimutant 2 (E2)	This study
Mucor bainieri	YES14	Epimutant 3 (E3)	This study
Mucor bainieri	YES15	Epimutant 4 (E4)	This study
Mucor bainieri	YES16	Epimutant 5 (E5)	This study
Mucor bainieri	YES17	Epimutant 6 (E6)	This study
Mucor bainieri	YES18	Mutant 1 (M1)	This study
Mucor bainieri	YES19	Epimutant 7 (E7)	This study
Mucor bainieri	YES20	Epimutant 8 (E8)	This study
Mucor bainieri	YES21	Epimutant 9 (E9)	This study
Mucor bainieri	YES22	Revertant after passage 4 of Epimutant 1	This study
Mucor bainieri	YES23	Revertant after passage 4 of Epimutant 2	This study
Mucor bainieri	YES24	Revertant after passage 12 of Epimutant 3	This study
Mucor bainieri	YES25	Revertant after passage 16 of Epimutant 4	This study
Mucor bainieri	YES26	Revertant after passage 12 of Epimutant 5	This study
Mucor bainieri	YES27	Revertant after passage 12 of Epimutant 6	This study
Mucor bainieri	YES28	Revertant after passage 28 of Epimutant 7	This study
Mucor bainieri	YES29	Revertant after passage 28 of Epimutant 8	This study
Mucor bainieri	YES30	Revertant after passage 24 of Epimutant 9	This study
Mucor pseudolusitanicus	CBS 540.78	Phylogenetic species 4	[1]
Ellisomyces anomalus	CBS 243.57	Phylogenetic species 5	[1]
Mucor atramentarius	CBS 202.28	Phylogenetic species 6	[1]
Mucor atramentarius	YES31	Mutant 1 (M1)	This study
Mucor atramentarius	YES32	Mutant 2 (M2)	This study
Mucor atramentarius	YES33	Mutant 3 (M3)	This study
Mucor atramentarius	YES34	Mutant 4 (M4)	This study
Mucor atramentarius	YES35	Mutant 5 (M5)	This study
Mucor atramentarius	YES36	Mutant 6 (M6)	This study
Mucor atramentarius	YES37	Mutant 7 (M7)	This study
Mucor atramentarius	YES38	Epimutant 1 (E1)	This study

Mucor atramentarius	YES39	Epimutant 2 (E2)	This study
Mucor atramentarius	YES40	Mutant 8 (M8)	This study
Mucor atramentarius	YES41	Revertant after passage 32 of Epimutant 1	This study
Mucor atramentarius	YES42	Revertant after passage 28 of Epimutant 2	This study
Mucor griseocyanus	CBS 223.56	Phylogenetic species 7	[1]
Mucor griseocyanus	CBS 116.08	Phylogenetic species 8	[1]
Mucor amethystinus	CBS 526.68	Phylogenetic species 9	[1]
Mucor lusitanicus	CBS 108.19	Phylogenetic species 10	[1]
Mucor variicolumellatus	CBS 236.35	Phylogenetic species 11	[1]
Mucor ramosissimus	CBS 135.65	Phylogenetic species 12	[1]
Mucor pseudocircinelloides	CBS 541.78	Phylogenetic species 13	[1]
Mucor circinelloides	CBS 192.68	Phylogenetic species 14	[1]
Mucor circinelloides	CBS 172.27	Phylogenetic species 15	[1]
Mucor circinelloides	CBS 394.68	Phylogenetic species 15	[1]
Mucor ctenidius	CBS 293.66	Phylogenetic species 16	[1]

Reference

1. Wagner, L., Stielow, J.B., de Hoog, G.S., Bensch, K., Schwartze, V.U., Voigt, K., Alastruey-Izquierdo, A., Kurzai, O., and Walther, G. (2020). A new species concept for the clinically relevant *Mucor circinelloides* complex. Persoonia 44, 67-97. https://doi.org/10.3767/persoonia.2020.44.03.

Table S2. Oligonucleotides utilized in this study.

Oligo Name	Sequence 5' to 3'	Description
JOHE53988/YES87	GGATCAGATGCTGCTTGCC	PS1 *fkbA* seq F
JOHE53989/YES88	ATGGGCTTGTTGCATCGC	PS1 *fkbA* seq R
JOHE54708/YES228	GCTCTTGATGCTGTGCTGTAC	PS1 *cnaA* seq F
JOHE54709/YES229	TGGTGTATGGTGTCGAAACTGA	PS1 *cnaA* seq R
JOHE54440/YES192	AGTTGATTGGACAAGCGCCT	PS1 *cnaB* seq F
JOHE54441/YES193	GTGCTCTGTTGTAATGCGATGG	PS1 *cnaB* seq R
JOHE54442/YES194	AGCACATGGTGGCTTGATTTC	PS1 *cnaC* seq F
JOHE54443/YES195	CAAGCAGCATCCATGTCTTGG	PS1 *cnaC* seq R
JOHE54443/YES196	TGTGCTCAACACAACATGTCG	PS1 *cnbR* seq F
JOHE54443/YES197	AGCTTGCTGAAGACTCACTCC	PS1 *cnbR* seq R
JOHE53990/YES89	GCCTACTTGAGCAAGCATCGT	PS3 *fkbA* seq F
JOHE53989/YES88	ATGGGCTTGTTGCATCGC	PS3 *fkbA* seq R
JOHE53557/YES31	CATTTGAGGATGCCAAGCGA	PS3 *cnaA* seq F
JOHE53558/YES32	TCAGCAGCTGCTCTTTTGC	PS3 *cnaA* seq R
JOHE53559/YES33	CATTTGAGGAAGCCAAGCGA	PS3 *cnaB* seq F
JOHE53560/YES34	GACCATCTTCTTCCTCCACAG	PS3 *cnaB* seq R
JOHE53561/YES35	ATCGCATGTATCGCAAGAGC	PS3 *cnaC* seq F
JOHE53562/YES36	AGGGATGTGGACTCGAGTTG	PS3 *cnaC* seq R
JOHE53563/YES37	CATGGAAGCTGACAAGGATGG	PS3 *cnbR* seq F
JOHE53564/YES38	GTTCTCGACCATGACCAGGA	PS3 *cnbR* seq R
JOHE53556/YES29	AACCGTGAGAAGATGACCCA	PS3 *actA* probe F
JOHE53557/YES30	TCCAAACGCAAGATAGCGTG	PS3 *actA* probe R
JOHE54916/YES253	GAAGCGAATGGACGACGAAT	PS3 positive control probe F
JOHE54917/YES254	AGAGCGTTAACTCTGTCCGT	PS3 positive control probe R
JOHE54857/YES231	ACATGGCCTTTGATTTGCCT	PS3 5' *fkbA* probe F
JOHE54858/YES232	AGGCCTATCATCATTCACTCTTGT	PS3 5' *fkbA* probe R
JOHE53457/YES18	GAATCTCTCCTGGCGATGG	PS3 *fkbA* probe exon2 F
JOHE53459/YES20	TCCCAACCCTTGATGACTTG	PS3 *fkbA* probe exon2 R
JOHE54914/YES251	TTGTCTGTTGGTGAGAAGGC	PS3 *fkbA* probe exon3 F
JOHE54915/YES252	GGGTAACCACGCTCACCATA	PS3 *fkbA* probe exon3 R
JOHE54859/YES233	ACTAAACTCCTTGCCTGGACA	PS3 3' *fkbA* probe F
JOHE54860/YES234	CAACCCTGCTACAGTAGATGC	PS3 3' *fkbA* probe R
JOHE56182/YES434	GAACGCTTCTTGGCTGAGAG	PS3 negative control probe F
JOHE56183/YES435	GTGGGCTGGATTGACTTCG	PS3 negative control probe R
JOHE56188/YES440	GAGGCAGCCCTACTCCTATG	PS3 *fkbA* near gene probe F
JOHE56189/YES441	ACGACGCTCATCGTTGGATA	PS3 *fkbA* near gene probe R
JOHE55723/YES333	TGAAGAGGAAGGCGATGCTT	*PS3_009593* probe F
JOHE55743/YES334	TCGGATGCAATGTGATTGGC	*PS3_009593* probe R
JOHE55830/YES365	TGCCCATACCTAATGGCAGT	*PS3_009595* probe F
JOHE55831/YES366	TTACTGCTGATGTGGCAGGT	*PS3_009595* probe R
JOHE55746/YES337	GCCACACATCAACCCACATT	*PS3_009596* probe F
JOHE55747/YES338	TCCAATGGAGGAGAGGAGGA	*PS3_009596* probe R
JOHE55748/YES339	GCCGAGAACAAGGCATCAAA	*PS3_009597* probe F

JOHE55749/YES340	GCGGTAGATGCTGTCTATCCA	*PS3_009597* probe R
JOHE55750/YES341	ACTGCCATCACTTGAAGCAC	*PS3_009598* probe F
JOHE55751/YES342	TTGTGAACCTTGCTGGATGC	*PS3_009598* probe R
JOHE55752/YES343	GCGACAGATCAAGCGTATCC	*PS3_009599* probe F
JOHE55753/YES344	GGCCTGCATCTTCGTTCTTT	*PS3_009599* probe R
JOHE53992/YES91	GCTGTGTCACGAAATGTGCA	PS6 *fkbA* seq F
JOHE53993/YES92	ATGGGATTGCTGCATCGC	PS6 *fkbA* seq R
JOHE53973/YES72	GGACTAGAAGACGCTCTCTCA	PS6 *cnaA* seq F
JOHE53974/YES73	GAGCCTGAGCTTGAGCTC	PS6 *cnaA* seq R
JOHE53975/YES74	GCCGACAACAAGCCATACTG	PS6 *cnaB* seq F
JOHE53976/YES75	GTCTTCACGGTGGCCTTC	PS6 *cnaB* seq R
JOHE53977/YES76	ATGCCAACATGTCTCACTCG	PS6 *cnaC* seq F
JOHE53978/YES77	CCCAATAGTAGCCTGACACAC	PS6 *cnaC* seq R
JOHE53979/YES78	CGTGTGCTTGTCAGGTTCAG	PS6 *cnbR* seq F
JOHE53980/YES79	ATGATGCTGAAACTCACGCG	PS6 *cnbR* seq R
JOHE54407/YES168	GGTGTTACCCACACTGTTCC	PS6 *actA* probe F
JOHE54408/YES169	CCAGCCATATCCAAACGCAA	PS6 *actA* probe R
JOHE54869/YES243	AATAGTCTTGTCTCATGCAGACT	PS6 positive control probe F
JOHE54870/YES244	GCACCAAGAGAGAGACGAG	PS6 positive control probe R
JOHE54865/YES239	AATGCACAAAGTCTCGGAGT	PS6 5' *fkbA* probe F
JOHE54866/YES240	TCCCTTCTTCGATCATGTGTCA	PS6 5' *fkbA* probe R
JOHE54405/YES166	GAATTGCTCCTGGTGATGGC	PS6 *fkbA* probe exon2 F
JOHE54406/YES167	TCGAGAGTACCAACGTAGTGG	PS6 *fkbA* probe exon2 R
JOHE54924/YES261	TCTGTTGGTGAGAAGGCCAA	PS6 *fkbA* probe exon3 F
JOHE54925/YES262	GGGTAACCACGTTCACCATA	PS6 *fkbA* probe exon3 R
JOHE54867/YES241	ACGTAAACCACCAAAGTCACC	PS6 3' *fkbA* probe F
JOHE54868/YES242	AGCTCAGATGTTTATGGCTTGC	PS6 3' *fkbA* probe R
JOHE56260/YES442	TCCGCAGAGGATCTTGAGAC	PS6 negative control probe F
JOHE56260/YES443	AGTAGACGGCGGGATGATTT	PS6 negative control probe R
JOHE56180/YES432	GCAAGTGATGCGATGTCTGA	PS6 *fkbA* near gene probe F
JOHE56181/YES433	CTCTTGCTGTGGGTTGGATG	PS6 *fkbA* near gene probe R
JOHE55756/YES347	GCTGCTGATACATCCAGTGC	*PS6_006517* probe F
JOHE55757/YES348	ATTGGAAGCAGCAGCTTGAG	*PS6_006517* probe R
JOHE55758/YES349	GCTGCCATGTTCTACGTTGT	*PS6_006518* probe F
JOHE55759/YES350	CGCCATCCAGGTAGTGTTTG	*PS6_006518* probe R
JOHE55760/YES351	TTGCAGCCGAGATGCAGATA	*PS6_006519* probe F
JOHE55761/YES352	TCCATGCTCGTTTGGAGATT	*PS6_006519* probe R
JOHE55762/YES353	GATAGAGCGCCACCATCAAC	*PS6_006520* probe F
JOHE55763/YES354	CTCTCAGGCTGAAACTGTGC	*PS6_006520* probe R
JOHE55764/YES355	GACTACGATGGCTGGGACAT	*PS6_006522* probe F
JOHE55765/YES356	CAGCTCGGCATAATGCTTCA	*PS6_006522* probe R
JOHE55766/YES357	TGGATAACACTCGCCAGGAA	*PS6_006523* probe F
JOHE55767/YES358	TCCTCTCTCTCTTGCTGCAC	*PS6_006523* probe R
JOHE55768/YES359	ATTGAAGAAGCCCGCGACAT	*PS6_006524* probe F
JOHE55769/YES360	CGACGAGAAGATGGTGGAGA	*PS6_006524* probe R
